# CD103^+^ dendritic cell–fibroblast crosstalk via TLR9, TDO2, and AHR signaling drives lung fibrogenesis

**DOI:** 10.1172/jci.insight.177072

**Published:** 2025-02-18

**Authors:** Hannah Carter, Rita Medina Costa, Taylor S. Adams, Talon M. Gilchrist, Claire E. Emch, Monica Bame, Justin M. Oldham, Steven K. Huang, Angela L. Linderholm, Imre Noth, Naftali Kaminski, Bethany B. Moore, Stephen J. Gurczynski

**Affiliations:** 1 Department of Microbiology and Immunology, University of Michigan, Ann Arbor, Michigan, USA.; 2 Section of Pulmonary, Critical Care and Sleep Medicine, Yale University School of Medicine, New Haven, Connecticut, USA.; 3 Division of Pulmonary and Critical Care Medicine, University of Michigan, Ann Arbor, Michigan, USA.; 4 Division of Pulmonary and Critical Care Medicine, University of California, Davis, California, USA.; 5 Division of Pulmonary and Critical Care Medicine, University of Virginia, Charlottesville, Virginia, USA.

**Keywords:** Immunology, Pulmonology, Dendritic cells, Fibrosis

## Abstract

Idiopathic pulmonary fibrosis (IPF) is characterized by progressive scarring and loss of lung function. With limited treatment options, patients die from the disease within 2–5 years. The molecular pathogenesis underlying the immunologic changes that occur in IPF is poorly understood. We characterize noncanonical aryl-hydrocarbon receptor (ncAHR) signaling in DCs as playing a role in the production of IL-6 and increased IL-17^+^ cells, promoting fibrosis. TLR9 signaling in myofibroblasts is shown to regulate production of TDO2, which converts tryptophan into the endogenous AHR ligand kynurenine. Mice with augmented ncAHR signaling were created by crossing mice harboring a floxed AHR exon 2 deletion (AHR_Δex2_) with mice harboring a CD11c-Cre. Bleomycin (blm) was used to study fibrotic pathogenesis. Isolated CD11c^+^ cells and primary fibroblasts were treated ex vivo with relevant TLR agonists and AHR-modulating compounds to study how AHR signaling influenced inflammatory cytokine production. Human datasets were also interrogated. Inhibition of all AHR signaling rescued fibrosis; however, AHR_Δex2_ mice treated with blm developed more fibrosis, and DCs from these mice were hyperinflammatory and profibrotic upon adoptive transfer. Treatment of fibrotic fibroblasts with TLR9 agonist increased expression of TDO2, and fibrotic fibroblasts activated IL-6 production in CD103^+^ DCs. Study of human samples corroborated the relevance of these findings in patients with IPF. We also show, for the first time to our knowledge, that AHR exon 2 floxed mice retain the capacity for ncAHR signaling.

## Abstract 2

Non-canonical AHR signaling in dendritic cells drives cytokine production in the fibrotic lung in response to ligands produced by fibroblasts.

## Graphical abstract

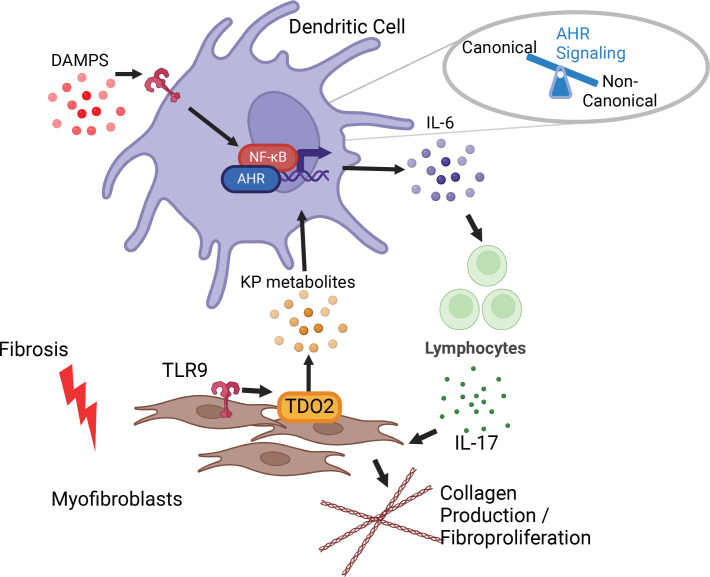

## Introduction

Idiopathic pulmonary fibrosis (IPF) is a progressive fibrotic disease with estimated survival of 2–5 years after diagnosis. Risk factors include male sex, cigarette smoking, and repeated viral infection, with median age at diagnosis of 66 years (1). Treatments slow progressive fibrosis, but only lung transplant is curative (2). Fibroblast activation and excess production of collagens are critical factors that underlie disease progression; however, fibrogenesis is a complex and poorly understood process requiring cooperation/crosstalk among multiple cell subsets. Myriad inflammatory cells accumulate in fibrotic lungs, including DCs, macrophages, and lymphocytes; however, the ultimate role of the immune system in initiation and progression of fibrosis is not understood. Immune responses may promote progressive scarring, but treating patients with steroids is counterintuitively harmful, highlighting the need for more research into the interactions between immune cell populations and fibrogenesis (3). We have previously defined an important role for tissue-resident DCs, and more specifically DCs expressing the aryl-hydrocarbon receptor (AHR), in virus-mediated pulmonary fibrosis following bone marrow transplantation (4, 5). Tissue-resident DCs have been shown to accumulate in the lungs of both IPF patients and bleomycin-treated (blm-treated) mice; however, their exact role in the initiation or progression of pulmonary fibrosis is unclear (6–8).

The canonical AHR pathway is an intracellular signaling cascade originally described as the major sensor of polycyclic aromatic hydrocarbon (PAH) toxicants, e.g., dioxin (9). More recently, endogenous AHR ligands such as the kynurenine family of tryptophan metabolites have been described (10, 11). Kynurenine can be made by indoleamine-2,3-dioxygenase (IDO) or tryptophan 2,3-dioxygenase (TDO), and kynurenine/tryptophan ratios are increased in patients with fibrotic lung disease (12). Increased kynurenine/tryptophan ratios in the blood are also prognostic indicators for many inflammatory lung diseases, including cancers and SARS-CoV-2 (13–18).

AHR is constitutively expressed in an inactive form sequestered in the cytoplasm bound to various chaperone proteins (19). Binding of ligand exposes a nuclear localization signal that facilitates translocation of AHR to the nucleus, wherein the chaperone coat is recycled and AHR dimerizes with its major canonical partner ARNT (20, 21). This AHR/ARNT dimer binds to xenobiotic response elements in the genome and activates transcription of a variety of gene products, including the CYP family of cytochrome P450 oxidases (22).

AHR is present and involved in inflammation in several lung cell types, including immune cells such as DCs, macrophages, and lymphocytes, as well as structural cells such as epithelial cells and fibroblasts. Recently, AHR has been studied for its interactions with other binding partners ARNT, i.e., NF-κB subunits RelA/RelB and KLF6, in what is termed “noncanonical AHR (ncAHR) signaling,” which is especially important in regulating expression of proinflammatory cytokines in immune cells such as DCs and macrophages (4, 23–25). Production of proinflammatory cytokines by DCs is augmented in the presence of AHR ligands, suggesting a role for AHR in concert with NF-κB when triggered by proinflammatory signals (26, 27). AHR and TLR, especially TLR9, have also been studied separately for their ability to promote fibrogenesis by directly augmenting fibroblast differentiation into profibrotic myofibroblasts (28, 29). Proinflammatory TLR9/AHR responsiveness has been linked to worsened IPF progression (30); however, the functional binding partners of AHR in inflammatory versus normal conditions, the triggers that divert AHR from its canonical pathway, and the exact role of AHR in complex pathologies such as fibrosis are not well understood.

Here, we present data indicating that ncAHR signaling is an important modulator of DC immune responses during the progression of pulmonary fibrosis. Using the bleomycin (blm) mouse model of pulmonary fibrosis, we demonstrate that AHR^+^ DCs accumulate in the lungs and drive production of IL-6 in an AHR-dependent fashion. AHR plays a role in differentiation of several immune cells, including Tregs, Th17 cells, and Th22 cells (31–33), which makes whole-body knockouts nonideal for studying immune responses. Using a conditional deletion of the DNA binding region of AHR (CD11c-Cre/AHR_Δex2_), we further demonstrate that deletion of canonical AHR function in DCs leads to significant upregulation of ncAHR signaling and proinflammatory cytokines and worsened fibrosis in the blm model. DCs have been primarily appreciated for their role in bridging adaptive and innate immunity, including through increasing Th17 differentiation in pulmonary fibrosis, a process itself mediated by AHR (34–36). The role of T cells in fibrosis is controversial, however, given the failure of corticosteroids to modulate disease (37). We demonstrate that adoptive transfer of ncAHR-activated DCs can promote fibrogenesis in an underappreciated CD4^+^ T cell–independent manner. We also provide evidence that myofibroblasts in the lungs of both IPF patients and fibrotic mice express TDO2, an enzyme that converts tryptophan into endogenous AHR ligands, and that specific inhibition of TDO2 ameliorates fibrosis. These data highlight an important role for TDO2 specifically in the fibrogenic circuit that is established between structural cells and immune cells in the fibrotic lung and provide evidence that TDO2 may be a viable druggable target for the treatment of pulmonary fibrosis.

## Results

### CD103^+^ DCs accumulate in the fibrotic lung, promote fibrogenesis, and show evidence of proinflammatory AHR signaling.

**Figure 1:**
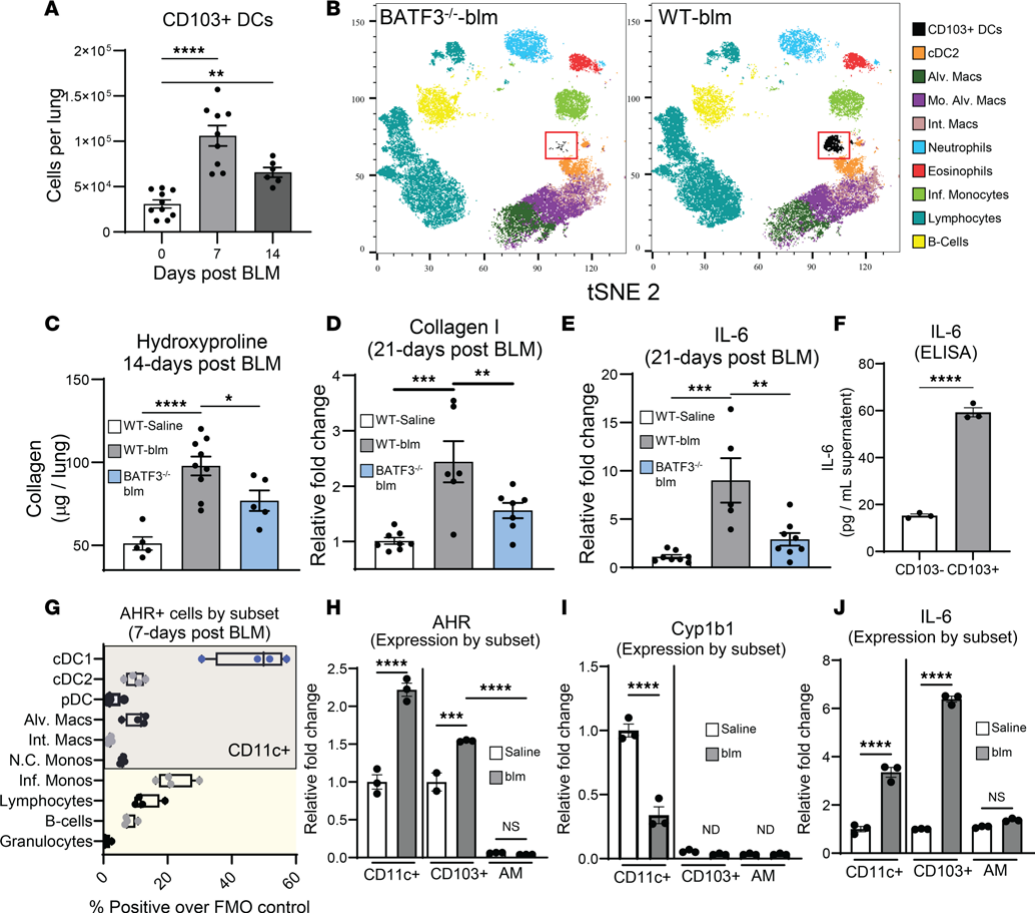
CD103^+^ DCs accumulate in the fibrotic lung and show evidence of proinflammatory AHR signaling. (**A** and **B**) Mice (*n* = 6–10 mice per group (**A**) and 5 per group (**B**) were treated with 0.75 U/kg blm for 0, 7, or 14 days (**A**) or 14 days (**B**). Lungs were harvested, and single-cell suspensions were analyzed by flow cytometry for the presence of CD103^+^ DCs (CD45^+^, CD11c^+^, MHC II^+^, CD24^+^, CD64^–^, CD103^+^) and lung leukocytes shown as a t-distributed stochastic neighbor embedding (tSNE) multidimensional reduction plot (full gating strategy in Supplemental Figure 1). Alv. Macs, alveolar macrophages; Mo. Alv. Macs, monocyte-derived alveolar macrophages; Int. Macs, interstitial macrophages; Inf. Monocytes, inflammatory monocytes. (**C**–**E**) Groups of mice (*n* = 5–9 mice per group [**C**] or *n* = 5–8 per group [**D** and **E**]) were treated with 0.75 U/kg blm for 14–21 days. Lungs were harvested, and collagen was quantified by hydroxyproline assay or qRT-PCR. (**F**) Blm-treated mice (*n* = 20 mice per group, pooled and analyzed in triplicate) were harvested 21 days following instillation. DCs were isolated from whole lung via a 2-step magnetic bead purification strategy using CD11c- and CD103-specific antibodies (CD11c^+^, CD103^–^ cells were retained and analyzed separately). After culturing for an additional 24 hours, expression of IL-6 in the supernatant was determined via ELISA. (**G**) Mice (*n* = 4 per group) were treated with 0.75 U/kg blm for 7 days, after which lungs were harvested and single-cell suspensions were analyzed via flow cytometry for AHR expression in the indicated cell subsets. (**H**–**J**) Mice (*n* = 5 per group) were treated with 0.75 U/kg blm or saline control (WT). Single-cell suspensions were prepared from pooled collagenase-digested lung tissue at 7 days after blm, and CD11c^+^ cells were purified via magnetic bead isolation. CD103^+^ DCs and AMs were purified via FACS from bulk CD11c^+^ magnetic sorted cells. Expression of the indicated transcripts was analyzed via qRT-PCR in each population. All experiments shown are representative of at least 3 independent experiments; statistical significance was determined via ANOVA or Student’s *t* test in **F** (*P < 0.05, **P < 0.01, ***P < 0.001, ****P < 0.0001).

We previously published that AHR^+^ CD103^+^ DCs were recruited to lungs, and drove lung injury and fibrosis following bone marrow transplantation and herpesvirus infection (4); however, a pathogenic role for AHR and DCs in noninfectious forms of pulmonary fibrosis is not known. To address this, we characterized lung DCs using a blm model of pulmonary fibrosis. Lung leukocytes were analyzed via flow cytometry at 7 and 14 days after treatment, and a significant increase in CD103^+^ DCs (CD45^+^, CD11c^+^, MHC II^+^, CD103^+^, CD11b^–^) was noted at both time points (Figure 1A). Differentiation of CD103^+^ DCs requires the transcription factor BATF3, and CD103^+^ DCs are completely absent in BATF3^–/–^ mice (38). To clarify the role of lung CD103^+^ DCs in mediating fibrogenesis, we treated BATF3^–/–^ mice with blm for 14 to 21 days and verified the specific absence of CD103^+^ DCs in the lung by flow cytometry (Figure 1B). BATF3^–/–^ mice exhibited durable protection from blm-induced pulmonary fibrosis, with less collagen production at 14 days as assessed by hydroxyproline assay and at 21 days as assessed by quantitative real-time PCR (qRT-PCR) for collagen I transcript (Figure 1, C and D). Interestingly, BATF3^–/–^ mice also displayed lower levels of IL-6 transcript, indicating that CD103^+^ DCs were important regulators of proinflammatory/fibrotic cytokine production following blm challenge (Figure 1E). We next analyzed expression of AHR and IL-6 in various lung immune cells via magnetic bead isolation or flow cytometry at 7 days after blm treatment (gating strategy for flow cytometry in Supplemental Figure 1; supplemental material available online with this article; https://doi.org/10.1172/jci.insight.177072DS1; markers for cell subsets detailed in Supplemental Table 2). Isolated CD103^+^ DCs produced increased amounts of IL-6 following blm instillation in comparison to lung CD11c^+^, CD103^–^ DCs (Figure 1F). The highest percentage of AHR^+^ cells was found in CD103^+^ DCs (conventional type 1 DCs [cDC1s], ~60%), while most other immune cells had AHR^+^ rates between 3% and 5% (Figure 1G). To further verify that expression of AHR was augmented in lung DCs, we isolated CD11c^+^ cells via magnetic bead isolation from the lungs of blm-treated or control mice and further purified 2 predominant CD11c^+^ cell populations — namely, alveolar macrophages (AMs) and CD103^+^ cDC1s (CD103^+^) — via FACS. CD11c^+^ cells from the lungs of blm-treated mice had increased expression of AHR in comparison to control mice but interestingly had decreased expression of the canonical AHR gene product Cyp1b1 (Figure 1, H and I). We further interrogated AMs and cDC1s for expression of these genes and found that CD103^+^ DCs expressed significantly more AHR than AMs (Figure 1H), but neither subset expressed appreciable amounts of Cyp1B1 (Figure 1I). In agreement with our previous data, CD11c^+^ cells isolated from blm-treated mice had increased expression of IL-6, which was further detected in a blm-specific manner in CD103^+^ DCs but was not detected in AMs, indicating that lung CD103^+^ cDC1s are an important source of proinflammatory cytokines (Figure 1J).

### Production of AHR ligands is augmented during blm-induced fibrosis, and complete AHR inhibition ameliorates disease.

**Figure 2:**
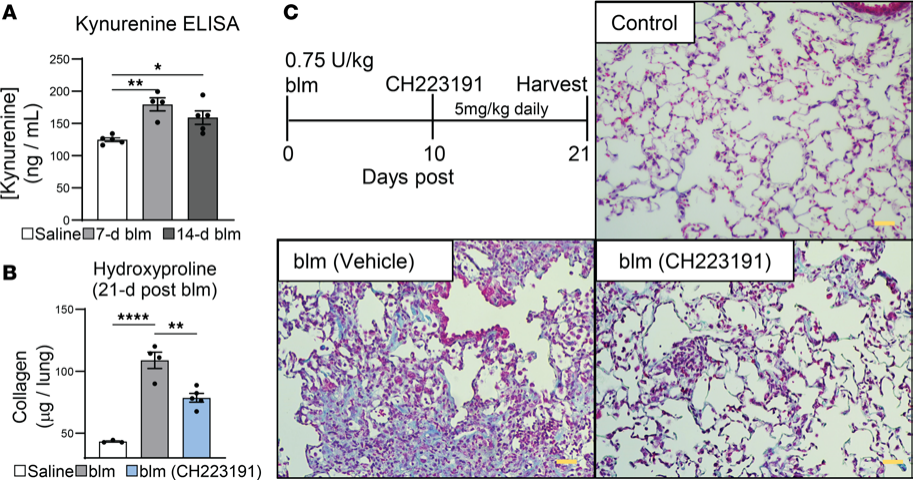
Production of AHR ligands is augmented during blm-induced fibrosis, and AHR inhibition ameliorates disease. (**A**) Mice (*n* = 4–5 per group) were treated with 0.75 U/kg blm. Lungs were harvested at the indicated times, and whole-lung homogenates were assayed for kynurenine concentration by ELISA. (**B**) Mice (*n* = 3–5 per group) were treated with 0.75 U/kg blm. Beginning on day 10, mice were treated daily with 5 mg/kg CH223191 dissolved in a vehicle of 2% Tween-20, 0.5% carboxymethylcellulose, in PBS or vehicle alone by oral gavage until day 18. Lungs were harvested on day 21, and lung collagen was quantified by hydroxyproline assay. (**C**) Representative histology of lungs from **B**. Sections were stained with trichrome, which highlights collagen fibers in blue (scale bars: 50 μM). All data are representative of at least 3 independent experiments; statistical significance was determined via ANOVA (*P < 0.05, **P < 0.01, ****P < 0.0001).

Several endogenous ligands for AHR have been described, most notably kynurenine, which is a metabolite of tryptophan processed by the heme-dependent oxygenases IDO1/2 and TDO2 (39). Whole-lung homogenates were prepared from saline-treated or blm-instilled mice. Concentrations of lung-associated kynurenine were assayed via ELISA, and a significant increase in lung-associated kynurenine was observed at both 7 and 14 days after blm treatment (Figure 2A). Furthermore, when blm-treated mice were given kynurenine by oral gavage during fibrogenesis, those mice had worse fibrosis and greater weight loss than did vehicle-treated controls, while canonical AHR signaling in the lung did not increase (Supplemental Figure 2). These data suggest that activation of AHR signaling in the fibrotic lung may promote development of blm-induced fibrosis. To test this hypothesis, we administered the AHR inhibitor CH223191 following blm instillation. Inhibition of AHR signaling ameliorated development of blm-induced pulmonary fibrosis. Mice treated with CH223191 exhibited less collagen deposition than nontreated mice (Figure 2B) and decreased evidence of fibrotic pathology on trichrome-stained lung sections (Figure 2C).

### Deletion of the AHR DNA binding domain reduces canonical AHR signaling in DCs, but AHR expression is retained and augments inflammatory noncanonical AHR signaling.

**Figure 3:**
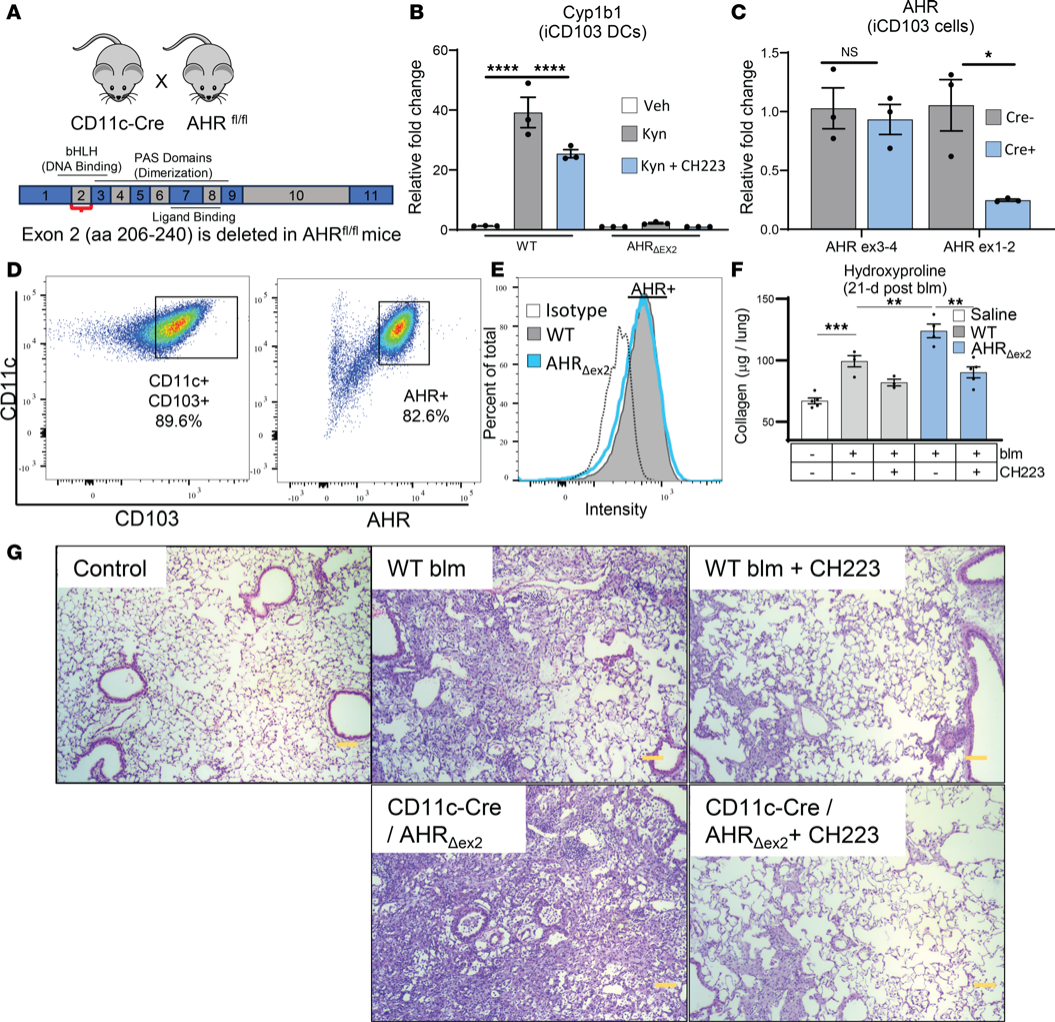
Deletion of AHR exon 2 reduces canonical AHR signaling in DCs, but AHR expression is retained and augments inflammatory ncAHR signaling. (**A**) Schematic of murine AHR depicting deletion of AHR exon 2 containing the DNA-binding basic helix-loop-helix (bHLH) domain. Numbered boxes correlate to individual exons. (**B** and **C**) iCD103 DCs were generated from WT B6 mice or AHR_Δex2_ mice and treated with 200 μM kynurenine (Kyn) or 10 μM CH223191 (CH223). Eighteen hours after treatment, RNA was harvested, and expression of the canonical AHR gene Cyp1b1 (**A**) or AHR (**B**) was analyzed via qRT-PCR. (**D**) iCD103 DCs from AHR_Δex2_ mice were analyzed via flow cytometry for expression of CD11c and CD103, as well as intracellular expression of AHR. (**E**) Representative histogram showing AHR expression in WT or AHR_Δex2_ iCD103 DCs analyzed via flow cytometry. (**F** and **G**) Mice (*n* = 5 per group) were treated with 0.75 U/kg blm. At 10 days after blm treatment, we administered CH223191 (5 mg/kg) or vehicle alone to the groups of mice daily until 18 days after blm treatment. Lungs were harvested at 21 days after blm, and collagen content was quantified via hydroxyproline assay (**F**); and lung fibrosis was examined via histopathological examination of fixed lung sections (scale bars: 100 μM in **G**). All data are representative of at least 3 independent experiments; statistical significance was determined via ANOVA (*P < 0.05, **P < 0.01, ***P < 0.001, ****P < 0.0001).

To further address AHR signaling in lung DCs, we bred mice that harbored floxed copies of AHR exon 2 (40) crossed to a strain that harbored Cre recombinase under control of the CD11c promoter (CD11c-AHR_Δex2_ mice, referred to as AHR _Δex2_; Figure 3A). Previous reports using these AHR exon 2 floxed mice described them as AHR-null mutants (40); however, we identified an alternative start codon (Supplemental Figure 3A, red text) that was uncovered during the fusion of AHR exon 1 and exon 3, resulting in a 12–amino acid N-terminal leader sequence (Supplemental Figure 3A, green text) fused in-frame to AHR exons 3–10 (Supplemental Figure 3A, blue text). DCs generated from these mice did not produce canonical AHR response genes following stimulation with kynurenine (Figure 3B); however, in AHR_Δex2_ mice there was no difference in the amounts of AHR transcript when primers specific for AHR exons 3–4 were used, while a significant reduction was observed when primers specific for the junction of exons 1–2 were used (Figure 3C). Furthermore, AHR was still detectable in CD103^+^ DCs produced from AHR_Δex2_ mice as assessed by intracellular flow cytometry (Figure 3, D and E).

We next generated cDNA from inducible CD103^+^ (iCD103) bone marrow DCs isolated from either heterozygous B6/AHR _Δex2_ or homozygous AHR_Δex2_ mice. PCR of the AHR ORF produced two bands in the heterozygous mice of approximately 2,400 and 2,200 bp (Supplemental Figure 3B), corresponding to the predicted sizes of the full-length AHR and AHR_Δex2_ ORF, respectively, and a single band of approximately 2,200 bp in the AHR_Δex2_ mice. We cloned these products into a pl-Cherry-NEO vector, which coexpressed each construct with mCherry via an internal ribosome entry site (IRES) sequence, and verified sequences of both full-length and AHR_Δex2_ PCR products via Sanger sequencing (Supplemental Figure 3C and data not shown). To verify the specificity of the AHR flow cytometry antibody, we conducted flow cytometry on 3T3 cells transfected with either full-length AHR or AHR_Δex2_ constructs and noted that approximately 30% of cells expressed mCherry after 48 hours with both constructs (Supplemental Figure 3D). We also examined expression of AHR in these transfected cells and noted an approximately 2-fold increase in AHR MFI in mCherry^+^ cells in comparison to mCherry^–^ cells in both full-length and AHR_Δex2_ transfections, indicating that both constructs produced an in-frame AHR protein (Supplemental Figure 3E). Western blot analysis of transfected cells with a C-terminal–specific AHR antibody showed that the AHR_Δex2_ produced a band of appropriate size, and we further queried for correct folding of the important protein-interacting domains PAS-A and PAS-B by AlphaFold (Supplemental Figure 3, F and G). Thus, we now report that although canonical AHR signaling was ablated following excision of exon 2, a truncated AHR protein was produced that mediated effects through the ncAHR pathway.

To assess the effect of DC AHR signaling on development of fibrosis, we administered blm to groups of WT B6 and AHR_Δex2_ mice. AHR_Δex2_ mice exhibited increased fibrosis and approximately 1.5-fold-increased collagen deposition versus WT mice 21 days after blm treatment (Figure 3, F and G). We were surprised by this result, as our initial experiment in which we used CH223191 to inhibit all AHR signaling ameliorated fibrosis (Figure 1C). We next administered CH223191 to groups of blm-treated WT or AHR_Δex2_ mice. Interestingly, inhibition of all AHR signaling was still ameliorative (Figure 3F), indicating that, while deletion of AHR exon 2 effectively ablated canonical AHR signaling in CD11c^+^ cells (Figure 3B), ncAHR signaling was likely still functional and could mediate profibrotic effects.

### Loss of canonical AHR signaling in CD11c^+^ cells exacerbates fibrosis via a ncAHR-dependent mechanism.

**Figure 4:**
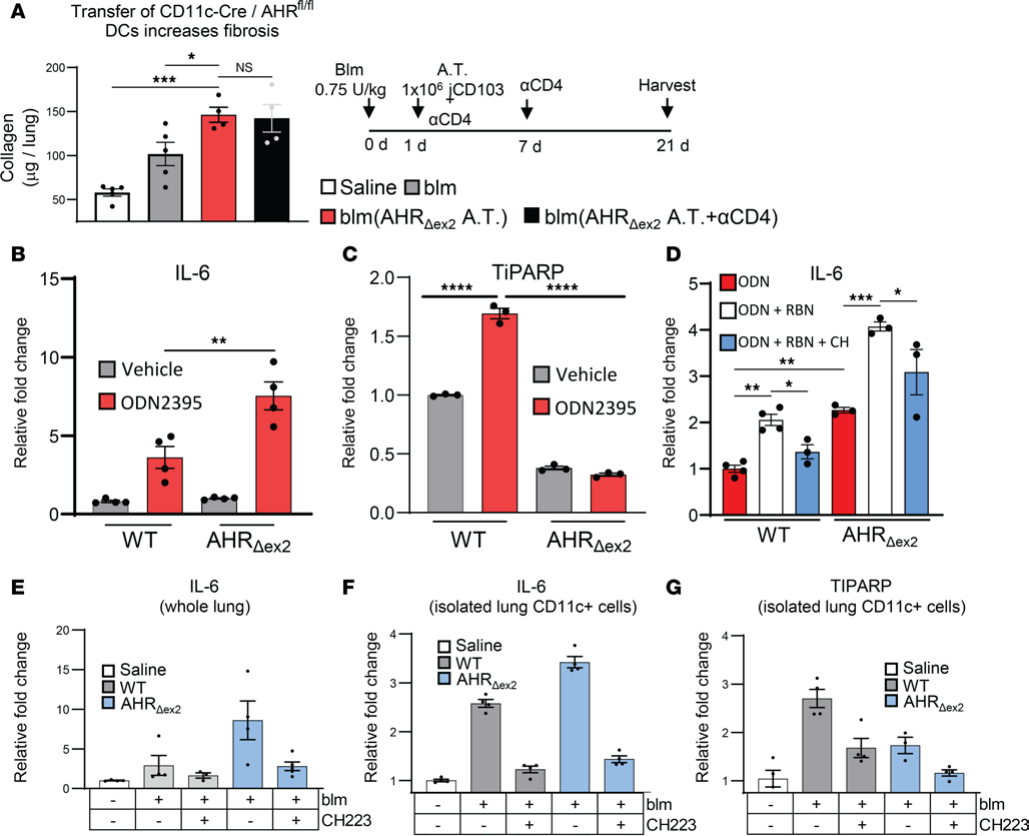
Loss of canonical AHR signaling in CD103^+^ DCs exacerbates fibrosis via a ncAHR-dependent mechanism. (**A**) Mice (*n* = 4–5 mice per group) were treated with 0.75 U/kg blm. At 1 day after blm treatment, 1 × 10^6^ iCD103 cells generated from naive CD11c-Cre^+^ AHR_Δex2_ mice (AHR_Δex2_ A.T.) were adoptively transferred via tail vein injection. Additionally, we administered 100 μg αCD4 neutralizing antibody to one group at 1 and 7 days after blm treatment. Lungs were harvested 21 days after blm, and collagen content was quantified via hydroxyproline assay. (**B**–**D**) iCD103 cells were generated as described. Cells were stimulated with 1 μM ODN2395 (TLR9 agonist), 10 μM RBN2397 (TiPARP inhibitor [RBN]), 10 μM CH223191 (AHR antagonist [CH]), or a vehicle control for 18 hours. Cells were harvested, and RNA was analyzed for expression of IL-6 or TiPARP transcript via qRT-PCR. (**E**) WT B6 or AHR_Δex2_ mice were treated with 0.75 U/kg blm, and CH223191 (CH223) was administered from day 10 to day 18 after blm treatment. Lungs were harvested at 21 day after blm, and expression of IL-6 transcript was quantified via qRT-PCR. (**F** and **G**) Mice (*n* = 5–7 per group) were treated with 0.75 U/kg blm or saline control (WT). Single-cell suspensions were prepared from collagenase-digested lung tissue at 7 days after blm treatment, and CD11c^+^ cells were purified via magnetic bead isolation, after which cells were pooled and treated overnight ex vivo (*n* = 4 per group) with either 30 μM CH223191 (CH223) or DMSO vehicle control. Expression of the indicated transcripts was analyzed via qRT-PCR. All data are representative of at least 3 independent experiments; statistical significance was determined via ANOVA (*P < 0.05, **P < 0.01, ***P < 0.001, ****P < 0.0001).

To further test the role of AHR in CD103^+^ DCs, we generated pure populations of iCD103 DCs from bone marrow stem cells (41). We previously published that iCD103 DCs express many cDC1 markers and express AHR, and are thus a suitable model for the study of cDC1 function in vitro, as the small number of cDC1s present in the lung at any given time makes isolation of significant numbers of these cells ex vivo difficult (4). We addressed the role of CD103^+^ DCs in mediating fibrosis by adoptively transferring iCD103 DCs from AHR_Δex2_ mice into groups of blm-treated WT mice. Lungs were harvested at 21 days after blm, and collagen content was assessed via hydroxyproline assay. Blm-treated B6 mice that had received an adoptive transfer of AHR_Δex2_ iCD103 cells developed increased fibrosis, exhibiting significantly increased lung collagen relative to mice receiving a sham transfer or a transfer of Cre^–^ littermate control iCD103 cells (Figure 4A and Supplemental Figure 4A). Along with adoptive transfer of AHR_Δex2_ iCD103 cells, we also administered neutralizing αCD4 antibody to blm-treated mice; however, no change in fibrosis was observed, indicating that the profibrotic effects of AHR_Δex2_ CD11c^+^ cells are independent of CD4^+^ T cells (Figure 4A).

The role of AHR in modulating cytokine responses is complex. Genes such as TiPARP that are induced through the canonical AHR pathway downregulate cytokine and interferon responses through posttranslational modification of critical kinases such as TBK1, thus shutting down NF-κB and INF regulatory factor (IRF) responses (42–45). In contrast, AHR itself can directly bind NF-κB and drive proinflammatory cytokine responses (27, 46–48). Multiple TLRs, including TLR9, have been implicated in the progression of pulmonary fibrosis (29, 49–51). For this study, modeling the sterile damage that occurs in IPF, we focused on TLR9, which is associated with cell damage. To address whether DC IL-6 expression was dependent on cooperation between AHR and TLR9 signaling, we stimulated iCD103 DCs with the TLR9 agonist ODN2395. Cells generated from AHR_Δex2_ mice expressed over 2-fold more IL-6 in comparison to ODN2395-treated iCD103 cells from WT mice (Figure 4B). Interestingly, while ODN treatment stimulated expression of TiPARP in WT iCD103 DCs, this effect was abrogated in AHR_Δex2_ iCD103 DCs (Figure 4C). Together, these data indicate that expression of TiPARP is dependent on canonical AHR signaling, while expression of IL-6 is augmented by ncAHR signaling. To further clarify the roles of both canonical and noncanonical AHR signaling in the production of IL-6, we treated iCD103 cells from WT or AHR_Δex2_ mice with TLR9 agonist and either the TiPARP inhibitor RBN2397 or a combination of RBN2397 and the AHR inhibitor CH223191. As previously observed, ODN-treated iCD103 cells from AHR_Δex2_ mice produced approximately 2-fold more IL-6 than WT cells (Figure 4D). Expression of IL-6 was increased by approximately 2-fold in both WT and AHR_Δex2_ cells when TiPARP was inhibited, indicating that TiPARP could significantly decrease production of inflammatory IL-6 (Figure 4D). Interestingly, inhibition of AHR with CH223191 was effective at ameliorating IL-6 production in both WT and AHR_Δex2_ ODN plus RBN2397–treated cells, indicating that ncAHR signaling was still functional in AHR_Δex2_ cells that had lost canonical AHR function (Figure 4D).

We next examined levels of IL-6 in blm-treated WT or AHR _Δex2_ mice that had been treated with the complete AHR inhibitor CH223191. In agreement with the increased IL-6 we observed in iCD103 DCs, AHR_Δex2_ mice exhibited increased expression of IL-6 both in whole lung and in CD11c^+^ cells isolated by magnetic bead purification from single-cell lung digestions (Figure 4, E and F). Expression of IL-6 was diminished by AHR inhibition (CCH223191) both at the whole-lung and CD11c^+^ cell level, indicating that AHR in CD11c^+^ cells directly potentiates IL-6 production in the fibrotic lung (Figure 4, E and F). We also verified that expression of TiPARP was similarly downregulated in lung CD11c^+^ cells from AHR _Δex2_ mice (Figure 4G) and thus conclude that the increased IL-6 observed was a product of the loss of antiinflammatory canonical AHR signaling and a concomitant increase in proinflammatory ncAHR signaling. Furthermore, given that lung cDC1s expressed significantly more AHR and IL-6 in comparison to CD11c^+^ AMs in blm-treated lungs (Figure 1, H and J), and AMs did not exhibit AHR-dependent modulation of proinflammatory IL-6 (Supplemental Figure 4B), we conclude that the effect of the CD11c-Cre was localized to lung DCs and not AMs.

### DC ncAHR signaling increases IL-17^+^ lymphocyte populations.

**Figure 5:**
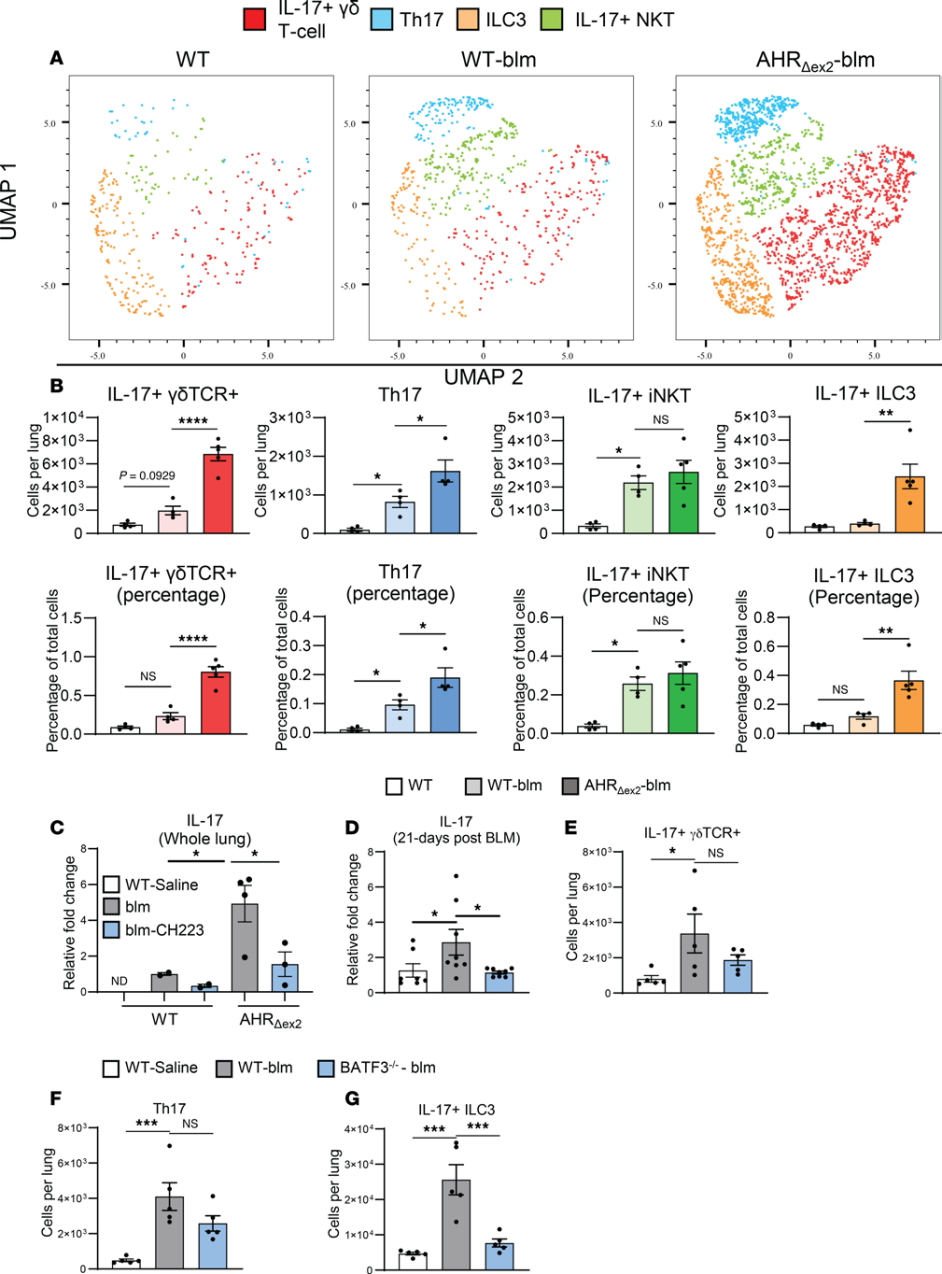
ncAHR signaling in CD103^+^ DCs drives IL-17 production from ILC3 in the lung. (**A**) WT B6 or AHR_Δex2_ mice (*n* = 3–5 per group) were treated (oropharyngeal delivery) with 0.75 U/kg blm. Seven days after blm treatment, lungs were harvested, and lung leukocytes were analyzed by flow cytometry for the following populations: Th17 (CD45^+^, Thy1.2^+^, CD3^+^, CD4^+^, IL-17A^+^), IL-17^+^ γδ T cell (CD45^+^, Thy1.2^+^, CD3^+^, CD4-, γδ-TCR^+^, IL-17A^+^), IL-17^+^ ILC3 (CD45^+^, Thy1.2^+^, CD3^–^, CD4^–^, IL-17^+^), IL-17A^+^ iNKT (CD45^+^, Thy1.2^+^, CD4^–^, CD3^+^, γδ TCR^+^, IL-17A^+^). (**B**) Quantification of cell populations identified in **A**. (**C**) WT B6 or AHR_Δex2_ mice were treated with 0.75 U/kg blm, and CH223191 (CH223) was administered from day 10 to day 18 after blm. Lungs were harvested at 21 days after blm treatment, and expression of IL-17 transcript was quantified via qRT-PCR. (**D**) Mice (*n* = 5–8 per group) were treated with 0.75 U/kg blm. Twenty-one days after blm treatment, lungs were harvested, and expression of IL-17 transcript from whole lung was analyzed via qRT-PCR. (**E**–**G**) Mice (*n* = 5 per group) were treated with 0.75 U/kg blm. Fourteen days after treatment, lung leukocytes were isolated via collagenase digestion and analyzed for the indicated cell population by flow cytometry: IL-17 γδ TCR^+^ (Thy1.2^+^, CD3^+^, CD4/8^–^, γδ TCR^+^, IL-17^+^), Th17 (Thy1.2^+^, CD3^+^, CD4^+^, IL-17^+^), ILC3 (Thy1.2^+^, CD3-, CD4/8^–^, IL-17^+^). All data are representative of at least 3 independent experiments; statistical significance was determined via ANOVA (*P < 0.05, **P < 0.01, ***P < 0.001, ****P < 0.0001).

Given that AHR_Δex2_ mice developed increased fibrosis and that ex vivo generated DCs produced increased IL-6 in response to TLR9 stimulus, we next examined whether AHR_Δex2_ mice would overproduce IL-17 in response to blm. We generated single-cell suspensions from lungs at 7 days after blm and analyzed expression of IL-17 in lymphocyte populations via flow cytometry. Flow cytometric analysis revealed that AHR_Δex2_ mice had increases in multiple IL-17^+^ lymphocyte populations, including conventional Th17 cells, IL-17A–producing γδ T cells, and putative IL-17A^+^ iNKT cells and ILC3s (Figure 5A, quantified in Figure 5B). Although we determined that CD4^+^ lymphocytes were not responsible for increased fibrosis (Figure 4A), we noted that the largest increase in IL-17A–expressing lymphocytes was in CD4^–^ γδ TCR^+^ and IL-17 producing ILC3 populations, which would not be affected by CD4 depletion. AHR_Δex2_ mice expressed approximately 5-fold more IL-17 at 21 days after blm treatment, and expression of both IL-6 and IL-17 was still susceptible to inhibition of AHR via administration of CH223191 (Figure 4, E and F, Figure 5C, and Supplemental Figure 5A), indicating that DCs and macrophages in CD11c-Cre/AHR_Δex2_ mice still had functional ncAHR signaling. To further elucidate the role of IL-17A in AHR-mediated pathogenesis, we gave anti–IL-17 neutralizing antibody to groups of AHR_Δex2_ or littermate control mice every 7 days starting at 3 days after blm treatment. Corroborating our observation that AHR_Δex2_ developed significantly more fibrosis than littermate controls, we observed nearly 40% mortality in AHR_Δex2_ mice that received vehicle injections in comparison to no mortality in littermate control mice at 21 days after blm treatment (Supplemental Figure 5B). AHR_Δex2_ mice that received anti–IL-17 therapy were completely protected from death (Supplemental Figure 5B), indicating that the increased IL-17 expressed in AHR_Δex2_ mice, where ncAHR signaling was bolstered, was a potent mediator of mortality.

We further verified the involvement of CD103^+^ DCs by examining expression of IL-17 in the lungs of blm-treated BATF3^–/–^ mice and found that loss of CD103^+^ DCs almost completely abrogated lung IL-17 responses — with the largest effect in IL-17–producing ILC3 and smaller/not statistically significant effects in IL-17–producing γδ T cells and conventional Th17 cells (Figure 5, D–G). We found the effects on IL-17 production in BATF3^–/–^ mice striking, as CD103^+^ DCs are more well known for their role in cross-presentation and activation of CD8^+^ T cells (52, 53). However, we analyzed populations of both lung-resident conventional CD8^+^ lymphocytes and IFN-γ^+^ Tc1 cells via flow cytometry and found no difference between WT and BATF3^–/–^ mice following blm treatment, while numbers of CD4^+^ lymphocytes and CD4/CD8 double-negative Thy1^+^ lymphocytes, i.e., ILCs and γδ T cells, were significantly reduced (Supplemental Figure 6).

### Fibroblasts from fibrotic lungs express kynurenine-producing enzymes in response to TLR9 stimulus and mediate IL-6 production from CD103^+^ DCs.

**Figure 6:**
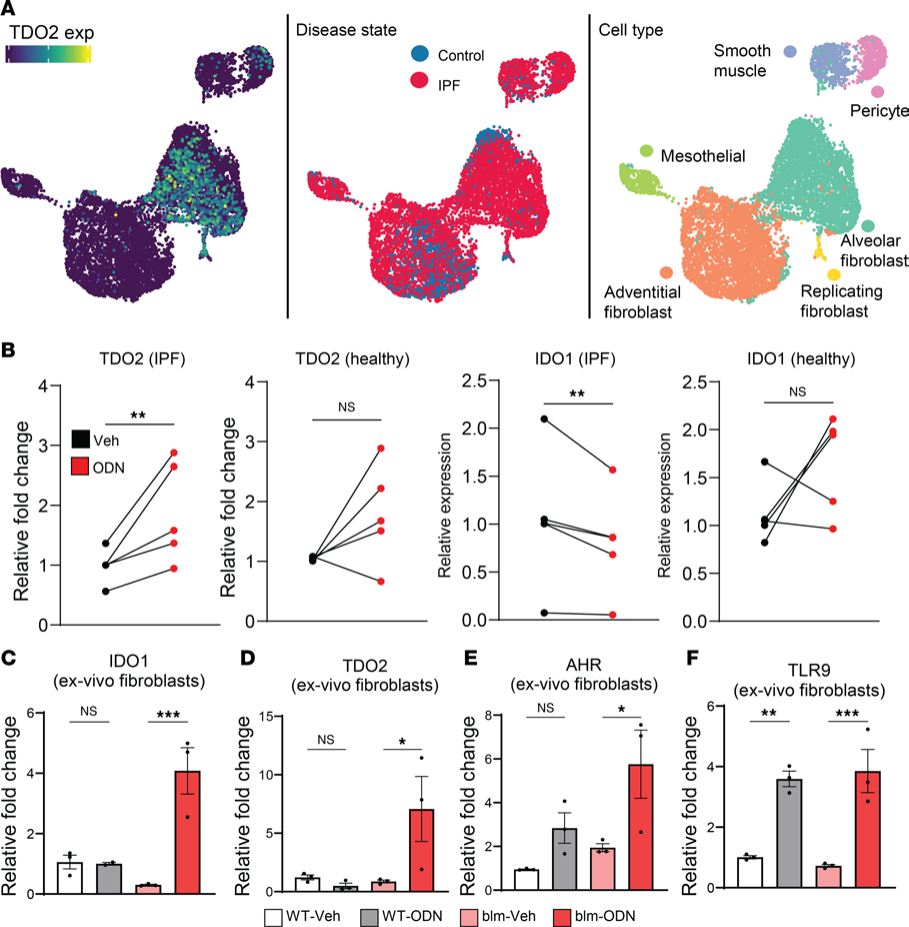
Fibroblasts isolated from fibrotic individuals express the kynurenine pathway enzymes IDO and TDO in response to TLR9 stimulation. (**A**) Uniform manifold approximation and projections (UMAPs) of human lung stromal cells labeled by normalized expression of TDO2, cell type, and disease. (**B**) Lung fibroblasts from patients with IPF (*n* = 5) or healthy control patients (*n* = 5) were treated with 100 μM ODN2395 (ODN) or vehicle (Veh) control for 18 hours, after which expression of either TDO2 or IDO1 was detected via qRT-PCR. (**C**–**F**) Lung fibroblasts were cultured from groups of mice treated with blm for 21 days or untreated as control. Fibroblasts were subsequently treated with the TLR9 agonist ODN2395 or vehicle control for 18 hours. Expression of the indicated transcript was analyzed via qRT-PCR. All data are representative of at least 2 independent experiments; statistical significance was determined via ANOVA (*P < 0.05, **P < 0.01, ***P < 0.001).

Endogenous AHR ligands are largely produced by the metabolism of tryptophan along the kynurenine pathway by enzymes such as IDO1 and TDO2. To determine what cells produce these enzymes in fibrotic lungs, we investigated data from 2 previously published single-cell RNA-Seq studies of IPF (54, 55). More than 9,000 stromal cells from 36 control and 44 IPF lungs were collectively analyzed and reclassified based on the markers and nomenclature described in Adams et al. (55). Expression of TDO2 was observed to be upregulated specifically in IPF AMs/myofibroblasts across both datasets; however, IDO1 was undetected in any stromal cell subset (Figure 6A and Supplemental Figure 7, A and B).

**Figure 7:**
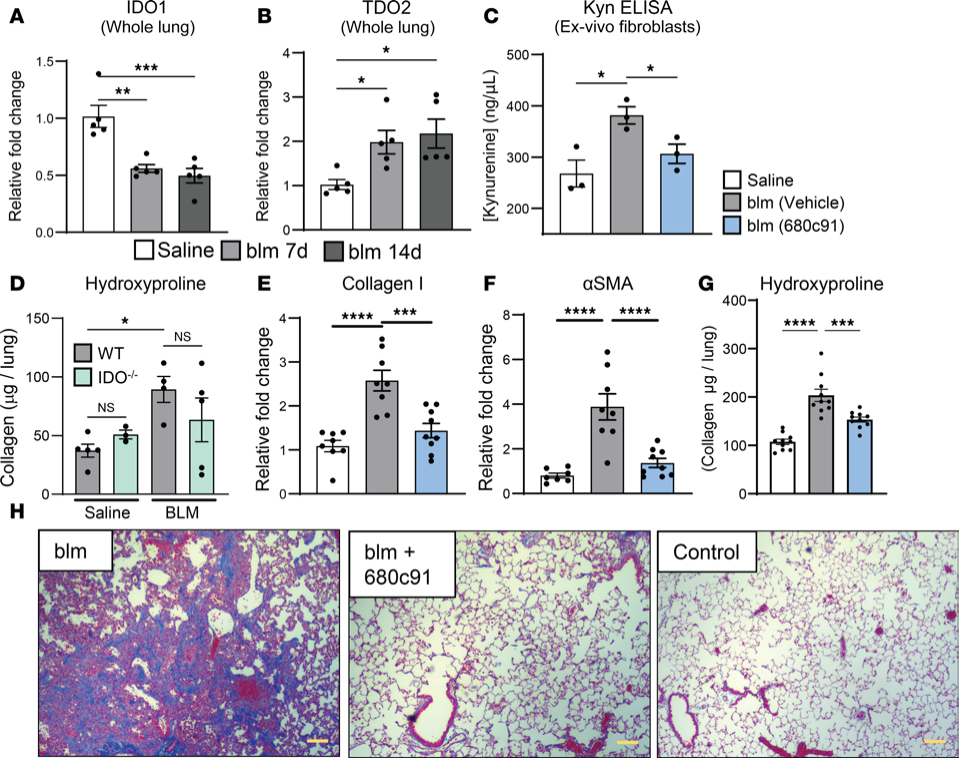
Inhibition of TDO2 rescues blm-induced fibrosis, but loss of IDO1 does not. (**A** and **B**) Groups of mice (*n* = 5 per group) were treated with 0.75 U/kg blm or saline control for 7–14 days, after which expression of the indicated transcript was analyzed in whole lung via qRT-PCR. (**C**) We administered 0.75 U/kg blm to the groups of mice (*n* = 3–5 per group). Lungs were harvested at 21 days after blm and assayed for collagen content via hydroxyproline assay. (**C**) Mice were treated with 0.75 U/kg blm or saline control for 21 days. Lung fibroblasts were isolated via crawl-out method and treated in triplicate with 100 μM tryptophan and 10 μM 680c91 (TDO2 inhibitor) or vehicle control for 18 hours after which supernatants were analyzed for the concentration of kynurenine by ELISA. (**D**) Groups of mice (*n* = 3–5 per group) were administered 0.75 U/kg blm. Lungs were harvested at 21 days after blm and assayed for collagen content via hydroxyproline assay. (**E** and **F**) Group of mice (*n* = 8–9 per group) were treated with 0.75 U/kg blm or saline control. Beginning on day 10 one group was treated with 15 mg/kg TDO2 inhibitor (680C91) daily by oral gavage until day 18, after which lungs were harvested and expression of the indicated transcript was assayed via qRT-PCR. (**G**) Mice (*n* = 9–10 per group) were treated with blm for 21 days and treated with either the TDO2 inhibitor or vehicle control from day 10 to day 21 via oral gavage. Lung collagen content was assessed on day 21 via hydroxyproline assay. (**H**) Representative histology (*n* = 2 mice per group) showing assessment of collagen deposition by trichrome staining (blue); scale bars: 100 μm. All data are representative of at least 2 independent experiments, statistical significance was determined via ANOVA (*P < 0.05, **P < 0.01, ***P < 0.001, ****P < 0.0001).

In addition to myeloid cells, TLR9 activation in fibroblasts has also been shown to be important in driving fibrosis (29, 56). Thus, we next analyzed expression of both TDO2 and IDO1 in explant lung fibroblasts from either patients with IPF or healthy control donors. Matching what was observed in our reanalysis of the Haberman et al. single-cell database, fibroblasts from patients with IPF showed a significant upregulation of TDO2 following stimulation with the TLR9 ligand ODN2395, while expression of IDO1 was significantly downregulated (Figure 6B). We further confirmed this phenotype in isolated fibroblasts from mouse lungs on day 21 after blm treatment. Expression of both IDO1 and TDO2 increased in ODN2395-treated fibroblasts from blm-treated mice but not in ODN2395-treated control fibroblasts (Figure 6, C and D). This effect was specific to TLR9 activation, as treating fibroblasts directly with IL-6 did not lead to upregulation of either IDO1 or TDO2 (Supplemental Figure 7C). AHR was also upregulated in the same conditions (Figure 6E); however, TLR9 was upregulated with ODN2395 regardless of blm treatment (Figure 6F). We further assayed for expression of IDO1 and TDO2 in whole lung following blm treatment. Expression of IDO1 was decreased at both 7 and 14 days after blm (Figure 7A); however, TDO2 expression was significantly increased at both time points (Figure 7B). Thus, it appears that TDO2 is preferentially induced over IDO1 in pulmonary fibrosis in vivo. We further confirmed that fibroblasts isolated from fibrotic mice produced more kynurenine than control fibroblasts, which was dependent on TDO2, as treatment with the TDO2 inhibitor 680c91 decreased kynurenine production back to the level in control fibroblasts (Figure 7C). To address the importance of TDO2 during fibrosis, we treated groups of mice with 680C91 and utilized IDO^–/–^ mice. Loss of IDO was not protective (Figure 7D and Supplemental Figure 8); however, inhibition of TDO2 significantly decreased both collagen mRNA expression and deposition and expression of the myofibroblast marker α–smooth muscle actin (α-SMA) during blm-induced pulmonary fibrosis, as well as fibrotic pathology, as assessed by trichrome staining of lung sections (Figure 7, E–H).

**Figure 8:**
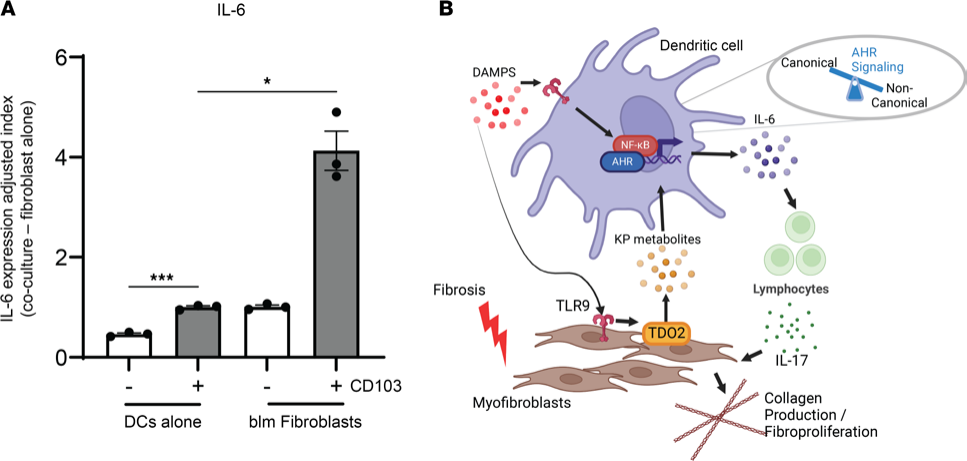
Ex vivo coculture of CD103^+^ DCs and fibroblasts increases IL-6 production. (**A**) CD103^+^ DCs were isolated using a 2-step magnetic bead isolation with CD11c- and CD103-specific antibodies. Cells were pooled from *n* = 20 mice and cocultured in triplicate with fibroblasts harvested from lungs of 21-day blm-treated mice. After 24 hours of coculture, RNA was isolated, and expression of IL-6 transcript was analyzed via qRT-PCR. Shown is a calculated index of IL-6 transcript expression minus the baseline expression of the fibroblasts without DC coculture in comparison to DCs alone (–, CD103-negative DCs; +, CD103-positive DCs). Statistical significance was determined via ANOVA (*P < 0.05, ***P < 0.001). (**B**) Myofibroblasts express the kynurenine pathway enzyme TDO2 in response to TLR9 stimulation. Kynurenine pathway (KP) metabolites activate ncAHR signaling in DCs, which, in cooperation with inflammatory TLR singling, augments production of IL-6, resulting in production of IL-17 from lymphocytes. Fibroblasts can be directly activated by IL-17, resulting in collagen production and fibroproliferation. DAMPs, damage-associated molecular patterns.

To solidify the observation that DCs interact with fibroblasts to drive proinflammatory and fibrotic responses, we cocultured primary CD103^+^ DCs from the lungs of blm-treated mice with fibrotic fibroblasts also isolated from blm-treated mice (7 and 21 days after blm instillation, respectively). In agreement with our previous data, CD103^+^ DCs alone produced more IL-6 than did CD103^–^ DCs (Figure 8A). IL-6 expression was further augmented by approximately 4-fold after 24- hours of ex vivo coculture with blm-treated fibroblasts (Figure 8A).

## Discussion

### Role of DCs in regulating pulmonary fibrosis.

The role of myeloid cells, especially DCs, in altering immune responses during development/exacerbation of pulmonary fibrosis is currently not well understood. Increased numbers of DC subsets infiltrate lungs of patients with IPF and mice with pulmonary fibrosis (8, 57–59). However, the study of DCs in pulmonary fibrosis has been overlooked due to publication of seminal work demonstrating that CD4^+^ and CD8^+^ lymphocytes, and by extension the DCs that prime those responses, are not important in mediating fibrotic effects (60). Recently, it is appreciated that DCs can modulate immune responses outside of antigen presentation through production of proinflammatory/fibrotic cytokines, including IL-6. IL-6 is a pleiotropic cytokine, is an early indicator of acute IPF exacerbation, and is involved in many aspects of DC biology. IL-6 produced from DCs can lead to dysregulated inflammation (61). We and others have demonstrated that DCs, especially CD103^+^ BATF3-dependent DCs, can prime and activate pathogenic IL-17^+^ lymphocyte responses through production of inflammatory cytokines such as IL-6 and TGF-β in innate lymphocytes as well as CD4^+^ T cells (5, 62–66). IL-6 has also been posited to act directly on type 2 pneumocytes, an effect we did not address in this study but may be playing a role (67). Here we demonstrate that CD103^+^ DCs infiltrate the fibrotic lung and mediate production of proinflammatory/fibrotic cytokines through cooperation of AHR and proinflammatory TLR signaling. We noted significant accumulation of CD103^+^ DCs in lungs of blm-treated mice beginning in the early inflammatory phase (7 days after treatment) and continuing into the fibrotic phase (14 days after blm; Figure 1A). Loss of CD103^+^ DCs in BATF3^–/–^ mice was protective, indicating a pathogenic role for lung-resident DCs (Figure 1, C–E).

### Role of ncAHR/NF-κB and TiPARP in modulating cellular cytokine responses.

The role of tryptophan metabolism and AHR signaling in mediating pulmonary fibrosis is also currently unclear. Kynurenine/tryptophan ratios increase in serum and lungs of patients with interstitial pneumonia, and treatment with antifibrotic pirfenidone significantly decreased levels of kynurenine, suggesting a positive correlation between tryptophan metabolism, AHR activation, and development of fibrosis (12, 68). We detected significantly elevated kynurenine from 7 to 14 days after blm treatment, corresponding to the early- to mid-fibrotic phase of blm-induced fibrosis (Figure 2A); and observed that, in agreement with our previously published study (4), CD103 cells that infiltrated the fibrotic lung expressed high levels of AHR but interestingly expressed low levels of canonical AHR targets such as Cyp1b1 (Figure 1). Canonical AHR signaling is immunosuppressive, and AHR target genes such as TiPARP can constrain production of interferons and proinflammatory cytokines via ADP-ribosylation of activators of NF-κB signaling such as TBK1 (42–45). Alternatively, ncAHR signaling is well characterized to bind NF-κB subunits such as RelA and RelB and enhance production of proinflammatory cytokines (27, 46–48). While others have found an antifibrotic role for AHR activation (69), we see that the addition of the AHR ligand kynurenine resulted in increased fibrosis in vivo, with no activation of canonical AHR signaling (Supplemental Figure 1). We also noted increased expression of IL-6 following blm treatment in both bead-isolated CD11c^+^ cells and FACS-purified CD103^+^ DCs, which correlated with increased expression of AHR but not canonical AHR gene products (Figure 1). Thus, even though CD11c^+^ cells expressed AHR and there was abundant AHR ligand produced in fibrotic lungs (Figure 2A), these cells did not activate antiinflammatory canonical AHR signaling and instead activated proinflammatory ncAHR signaling.

We generated mice that lacked AHR exon 2 in CD11c^+^ cells including pulmonary DCs and macrophages (Figure 3A and Supplemental Figure 3). Interestingly, while DCs produced from these mice lacked canonical AHR signaling (Figure 3B), we still detected AHR at the transcript and protein level (Figure 3, C–E, and Supplemental Figure 3). Furthermore, sequencing of cDNA generated from AHR_Δex2_ DCs indicated that an alternative start codon was formed when exon 2 was deleted and an AHR antibody specific for a C-terminal epitope was able to detect expression of AHR in cells transfected with a plasmid encoding the AHR_Δex2_ construct (Supplemental Figure 3, A, C, D, and F). Together, these data indicated that a truncated AHR product was expressed in AHR_Δex2_ and could likely still mediate ncAHR interactions. When we administered blm to AHR_Δex2_ mice, we noted increased fibrosis in comparison to control mice that was still AHR dependent (Figure 3, F and G), indicating that loss of canonical AHR signaling in CD11c^+^ cells resulted in increased ncAHR signaling that exacerbated pulmonary fibrosis. Interestingly, another group using a similar AHR exon 2 deletion mutant saw a similar aggravated pulmonary fibrosis phenotype when using a blm model (70). The authors attributed this phenotype to a complete loss of AHR function, but given our data, and the fact that even the original creators of the AHR-transgenic model state that there is a distinct possibility that truncated AHR proteins mediate noncanonical effects when just exon 2 is deleted (71), we must reinterpret these phenotypes through the lens of noncanonical versus canonical AHR signaling.

To determine whether the increased fibrosis seen in AHR_Δex2_ was intrinsic to DCs, we adoptively transferred ex vivo generated iCD103 DCs from AHR_Δex2_ mice into blm-treated B6 mice and noted that there was more fibrosis when DCs were transferred from CD11c-Cre/AHR_Δex2_ compared with Cre^–^ mice (Supplemental Figure 4A) or with sham transfer (Figure 4A and Supplemental Figure 4A). We further show that AHR could synergize with TLR9 activation and promote expression of IL-6 in DCs; however, expression of canonical AHR genes that limit cytokine production, e.g., TiPARP, was lost in AHR_Δex2_ DCs (Figure 4, B–D). Thus, blm-induced fibrosis resulted in increased expression of AHR; however, the signaling was shunted toward noncanonical signaling, resulting in increased expression of proinflammatory cytokines. It is currently unclear why ncAHR signaling is promoted over canonical signaling in fibrotic lungs; however, several transcription factors, such as HIF1α, that are upregulated during fibrogenesis bind to the canonical AHR partner ARNT (HIF1β) (72). Thus, it is plausible that dimerization of HIF1α-ARNT may reduce the available pool of ARNT, which promotes AHR–NF-κB dimerization; this would shift the balance of AHR signaling from canonical, antiinflammatory to noncanonical, inflammatory signaling, driving excessive fibrogenesis.

### Role of DC associated AHR signaling in fibrogenesis.

Due to the observed increase in lung kynurenine at 7–14 days following blm treatment, we opted to utilize pharmacologic inhibition of AHR at this later time point as opposed to inhibiting AHR from the start of blm installation. Complete inhibition of AHR signaling via administration of CH223191 reduced fibrosis when started late in the progression of disease (10 days after blm), indicating a role for pulmonary AHR signaling in promoting fibrogenesis (Figure 2, B and C). Counterintuitive findings from another study showed that exogenous administration of the AHR ligand FICZ ameliorated pulmonary fibrosis, presumably by augmenting numbers of FoxP3^+^ Tregs and decreasing IFN-γ^+^ CD4^+^ and IL-17A^+^ γδ^+^ T cells (73). However, this study did not look specifically at AHR signaling in pulmonary immune cells, nor did it demonstrate whether FICZ is produced during fibrosis. Furthermore, mice were treated directly after administration of blm on days 0, 1, and 2, making it difficult to determine direct effects of pulmonary AHR signaling in inhibiting fibrosis versus modulating early lung injury after blm treatment.

In contrast, we saw decreased numbers of FoxP3^+^ Tregs, Th17 cells, and IL-17A–producing ILC3s and γδ^+^ T cells when we inhibited all AHR signaling during active fibrogenesis (Supplemental Figure 5). We attribute this reduction in IL-17A expression to interactions between lymphocytes and CD103^+^ DCs, as we saw a similar reduction in IL-17A–expressing lymphocytes in blm-treated BATF3^–/–^ mice, which completely lack CD103^+^ pulmonary DCs, and decreased production of IL-6 in iCD103 cells treated with the AHR antagonist CH223191 (Figure 4, D and F, and Figure 5, E–G). The role of the IL-6/IL-17 axis is appreciated in the progression of pulmonary fibrosis. Depletion of either IL-6 or IL-17 is protective for blm-induced fibrosis, and we have previously published that IL-17 can act directly on fibroblasts to promote collagen production and fibroproliferation (5, 74–76). Even in patients with IPF, expression of IL-6 and some IL-17 isoforms is associated with more rapid time to death or lung transplantation (Supplemental Figure 9, A and B), and we further found that patients with IPF had significantly increased plasma levels of kynurenine in comparison to healthy controls (Supplemental Figure 9C).

Little is known about how CD103^+^ DCs may alter lymphocyte function in pulmonary fibrosis. Loss of circulating CD141^+^ DCs (human equivalent of murine CD103^+^ DCs) in the blood of patients with IPF is correlated with a worsened prognosis and increased production of cytokines, such as IL-6, which we and others have shown is predictive of increased severity of disease and lowered transplant-free survival time (Supplemental Figure 9) (77). We surmise that this loss of circulating DCs is a result of migration out of the blood and into the lung tissue, as other groups have shown accumulation of pulmonary DCs in IPF patients that correlates with disease severity (7, 8). Studies have suggested that outside of their normal role in presentation or cross-presentation of antigen to conventional CD4^+^ and CD8^+^ T cells, CD103^+^ DCs may physically interact with tissue-resident γδ^+^ T cells and ILC3s to modulate the expression of IL-17 (66, 78). Our data corroborate this, and we observed decreased numbers of IL-17^+^ ILC3s when CD103^+^ DCs were ablated using BATF^–/–^ mice (Figure 5G). Intriguingly, interactions between CD103^+^ DCs and nonclassical lymphocytes appear to involve sensing of the microbiota. While we did not analyze the lung microbiota in this study, alterations in the lung microbiota are correlated with the outcome and progression of pulmonary fibrosis in both animal models and in patients with IPF (30, 79), and the signaling axis between pulmonary DCs and the lung microbiota warrants further study.

### Fibroblasts produce AHR ligands in response to inflammatory stimulus in the fibrotic lung.

Interrogation of 2 previously generated single-cell datasets from patients with IPF indicated that pulmonary alveolar fibroblasts expressed the kynurenine-producing enzyme TDO2, while expression in normal lung fibroblasts was virtually nonexistent (Figure 6A) (54, 55). In agreement with this, we analyzed expression of TDO2 in isolated fibroblasts from both patients with IPF and blm-treated mice treated with the TLR9 agonist ODN2395, and saw significant upregulation in both (Figure 6, B and D). Interestingly, expression of IDO1 was significantly downregulated in ODN-stimulated IPF fibroblasts (Figure 6B) but was augmented in murine fibroblasts (Figure 6C). This discrepancy is likely a result of the isolation method used for murine fibroblasts, in that whole lungs were minced, which undoubtedly resulted in a heterogenous population of fibroblast subsets from both fibrotic and nonfibrotic areas of the lung. Reanalysis of single-cell sequencing data confirmed TDO2 expression in IPF patient myofibroblasts (Figure 6A); however, we did not detect appreciable expression of IDO1 in human myofibroblasts, and overall expression of IDO1 was shown to decrease in the lungs of fibrotic mice while expression of TDO2 increased (Figure 7, A and B).

Recently, it was reported that fibroblasts are a major source of IDO expression in the fibrotic lung and that IDO is protective for blm-induced fibrosis (69). However, it should be noted that this study cultured fibroblasts from primary tissue only for 24 hours, whereas standard practice for isolation of lung fibroblasts is much longer, i.e., 7 to generally 14 days, allowing for the death of immune cells and other short-lived cells that can express high levels of IDO and other immune mediators (80). Further in contrast to this study, we found that genetic deletion of IDO1 had no effect on fibrogenesis or expression of IL-6 (Figure 7D and Supplemental Figure 8), and we found no evidence of IDO expression in analysis of fibroblast subsets from patients with IPF (Supplemental Figure 7B), while treatment of mice and fibrotic fibroblasts with the specific TDO2 inhibitor 680C91 decreased kynurenine production, ameliorated fibrosis, and reduced collagen production and expression of the myofibroblast marker α-SMA (Figure 7). We highlight that 68C091 was found to have no cross-inhibitory activity with IDO1, and we conclude that the effects we observed were specific to TDO2 and not IDO1 (81).

Interestingly, we noted increased expression of AHR and TLR9 in blm-treated fibroblasts (Figure 6, E and F). Furthermore, the more-robust activation profiles of fibroblasts isolated from patients with IPF and exposed to TLR9 agonist correlated with increased AHR activation in matched myeloid cells (30), indicating that AHR plays a fundamentally profibrotic role. Expression of TLR9 is associated with rapid progression of IPF in patients, and loss of TLR9 signaling is protective from blm-induced fibrosis (29, 49, 50). Thus, we ultimately conclude that proinflammatory ncAHR activation that crosstalks with TLR signaling is an important driver of pulmonary fibrosis. Other studies have concluded that AHR plays a protective/antifibrotic role; however, it should be noted that many of the studies detailing an antifibrotic role for AHR in various cell types (69, 70) use the same AHR^fl/fl^ mice, characterizing them as complete AHR knockouts. We now conclude that these mice actually express an AHR construct that is hyperinflammatory through excessive ncAHR activation and interpretation of these studies must be reevaluated.

Our study and the work of others indicate that a fibrogenic circuit is established between TLR9^+^ fibroblasts and AHR^+^ myeloid cells that is important in progression of pulmonary fibrosis through modulation of profibrotic cytokines such as IL-6 and IL-17 (working model in Figure 8B). More work is needed to fully understand the switch to ncAHR signaling during fibrogenesis and to understand the spatial crosstalk between fibroblasts and DCs. Furthermore, our work does not examine the effect of kynurenine on cell types besides dendritic cells, including lung fibroblasts and epithelial cells, which have known roles for AHR. However, given the need for new treatments for patients with IPF, drugs that modulate kynurenine pathway enzymes, i.e., TDO2, or AHR directly represent an exciting possibility with untapped therapeutic potential.

## Methods

Further information is provided in Supplemental Methods.

### Sex as a biological variable.

Our study exclusively examined male mice. IPF is predominantly a disease affecting male patients. In line with this, female mice develop much-less-severe fibrosis in response to bleomycin.

### Mouse line, cell lines, and reagents.

Details about mouse strains and treatments are provided in Supplemental Methods.

### Generation of iCD103 DCs.

iCD103 DCs were generated from mouse bone marrow as previously described (4, 41).

### Fibroblast isolation.

Fibroblasts were isolated as previously described (80). In short, groups of mice were administered blm or saline as control. Twenty-one days after blm treatment, lungs were harvested and minced with scissors. Minced lungs were incubated in complete DMEM (DMEM + 10% FBS and 1% penicillin/streptomycin cocktail) for 7 days to let fibroblasts adhere. Medium was changed at 7 days and every 2 days afterward. Cells were trypsinized and plated on day 14 after isolation for subsequent assays.

Human fibroblasts were cultured from the outgrowths of lung tissue of patients with IPF derived from explanted lungs obtained at the time of transplantation, diagnosed as IPF by multidisciplinary conference prior to transplant, and confirmed to show a pathology of usual interstitial pneumonia after transplant. Control fibroblasts were cultured from nonfibrotic lungs obtained from deceased donors (Gift of Life, Michigan) whose lungs were deemed unsuitable for transplant.

### Single-cell RNA-Seq analysis.

Processed gene expression data from Habermann et al. (54) was downloaded from NCBI’s Gene Expression Omnibus database (GEO GSE135893). Raw sequencing data from Adams et al. (55) was downloaded from GEO (GSE136831) for reprocessing.

### Generation of single-cell suspensions and flow cytometry.

Single-cell suspensions were generated via collagenase digestion of lung tissue as previously described (84).

### Isolation of CD11c^+^ DCs from single-cell suspensions.

Primary lung DCs were isolated using either anti-CD11c beads (Miltenyi Biotec) or anti-PE/anti-FITC beads (STEMCELL) using CD11c-PE– and CD103-FITC–conjugated primary antibodies sequentially.

### Quantification of collagen via hydroxyproline assay.

Collagen was quantified as described previously (85).

### Isolation of RNA for qRT-PCR.

RNA was purified from lung tissue or cell pellets via incubation with TRIzol reagent following the manufacturer’s directions (a detailed list of primers used in this study is provided in Supplemental Table 1).

### Statistics.

Statistical analysis was conducted in GraphPad Prism v.9.1.0. Statistical significance was determined using 2-tailed Student’s *t* test when 2 groups were compared and 1-way ANOVA using Holm-Šidák’s post-test when 3 or more groups were compared. *P* values less than 0.05 were considered statistically significant. Relevant statistical information, i.e., *P* values and *n* values, is given in individual figure legends.

### Study approval.

All mouse experiments were conducted under an animal protocol approved by the University of Michigan IACUC. All patients provided written informed consent, and the protocol was approved by the University of Michigan Institutional Review Board (HUM00105694).

## Author contributions

HC performed experiments, analyzed data, and wrote and edited the manuscript. TSA and NK provided, analyzed, and interpreted single-cell RNA-Seq data. SKH, JMO, ALL, and IN provided clinical data and samples. BBM analyzed and interpreted data and edited the manuscript. SJG wrote and edited the manuscript, as well as analyzing and interpreting data.

## Supplementary Material

Supplemental data

Unedited blot and gel images

Supporting data values

## Version 1 02/18/2025

In-Press Preview

## Version 2 03/24/2025

Electronic publication
