# Supplementary material for: CD103+ dendritic cell–fibroblast crosstalk via TLR9, TDO2, and AHR signaling drives lung fibrogenesis
Source: JCI Insight. 2025 Feb 18;10(6):e177072. doi: 10.1172/jci.insight.177072 (PMC11949071; doi:10.1172/jci.insight.177072)
Supplement: Supplemental data [file jciinsight-10-177072-s192.pdf]

Supplemental Figure 1

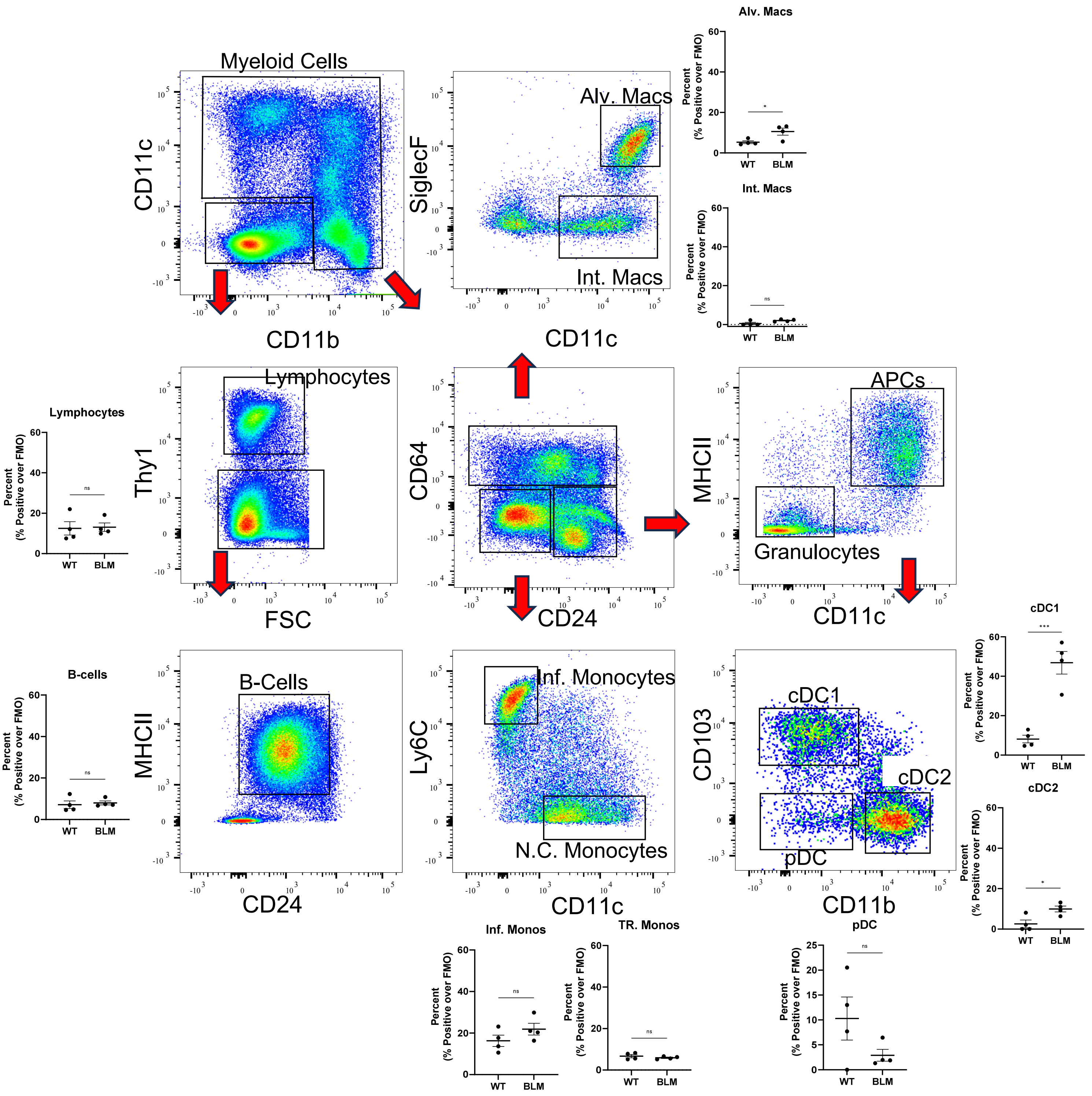

Supplemental Figure 1. Gating strategy for identification of AHR+ lung leukocytes. Mice (n = 4 per group) were treated with blm for 7-days or left untreated as control (WT). Single cells suspensions were generated from collagenase digested lung tissue and subpopulations of lung leukocytes were identified via flow cytometry. Cells were first gated as either CD11c+ CD11b+ (myeloid cells) or double negative. Double negative non-myeloid cells were further gated as Thy1+ (lymphocytes) or MHCII+, CD24+ B-Cells). Myeloid cells were further gated as CD64+ (Macrophages) or CD24 (Granulocytes and APCs). CD64+ macrophages were subdivided into CD11c+, SiglecF+ (Alveolar Macrophage) or SiglecF-, CD11c+ (Interstitial Macrophage). CD24+ cells were divided into MHCII+ CD11C+ CD103+ (cDC1), MHCII+, CD11C+, CD11B+ (cDC2), CD11c+, CD11B-, MHCII int (pDC) or MHCII-, CD11C- (Granulocytes). CD64-, CD24- cells were further gated into Ly6C+, CD11C- (Inflammatory Monocytes) or Ly6C-, CD11C+ (Non-Classical Monocytes). Expression of AHR was detected in all subpopulations via permeabilization of fixed cells in conjunction with intracellular antibody staining. AHR expression levels were verified using a flow minus one strategy (not shown). AHR expression levels of each subpopulation are depicted as graphs next to the relevant gated population.

Supplemental Figure 2

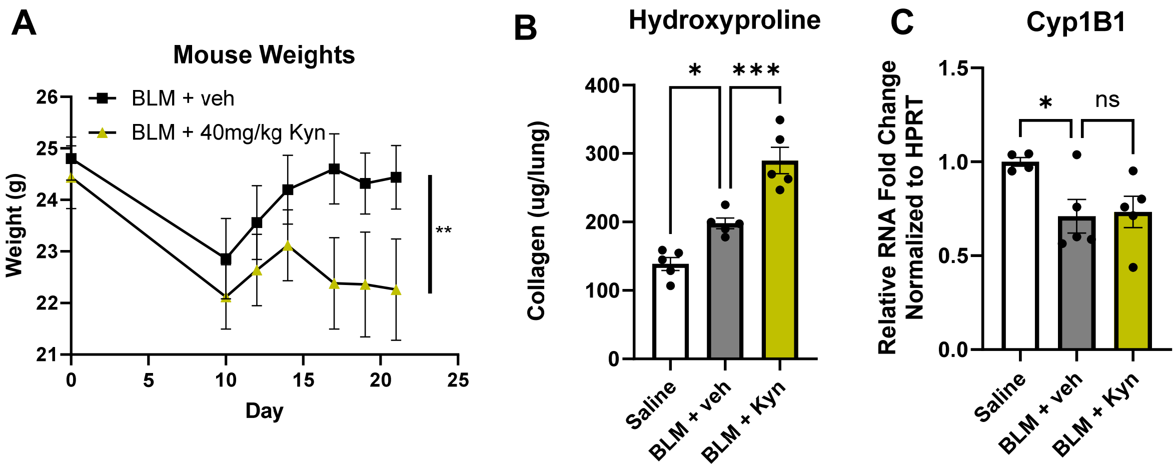

Supplemental Figure 2. Kynurenine treatment during fibrogenesis increases fibrosis. Mice (n = 5 per group) were treated with blm for 21-days or given saline as control. Blm-treated mice were given 40mg/kg of kynurenine by oral gavage daily d10-20 or a vehicle control and lungs were harvested on d21. Error bars represent SEM. A) Mouse weights over the time course of the experiment. ** = $P < 0.01$ by two-way ANOVA with Tukey test. B) Hydroxyproline assay measuring collagen levels in the harvested lungs at d21.* = $P < 0.05$, *** = $P < 0.001$ one-way ANOVA with Dunnett test. C) Relative expression levels of Cyp1B1 RNA transcript in whole lung by RT-qPCR determined by ΔΔCT method, normalized to HRPT transcript. * = $P < 0.05$ by one-way ANOVA with Dunnett test.

Supplemental Table 1

Table I. Primers and probes used for qRT-PCR

Gene Name		Primer Sequence
IL-17A	Forward	5'-CCGCAATGAAGACCCTGATAG-3'
	Reverse	5'-GCTTTCCCTCCGCATTGA-3'
	Probe	5'-GGGAAGCTCAGTGCCGCCAG-3'
Cyp1b1	Forward	5’-CAGAGCTTCTCCCAGATCCC-3’
	Reverse	5’-GGACTGTCTGCACTAAGGCT-3’
	Probe	5’-CGCTTCATCGCAGCATGGCCACC-3’
IL-6	Forward	5’-GACTTCCATCCAGTTGCCTTCT-3’
	Reverse	5’-CTGTTGGGAGTGGTATCCTCTGT-3’
	Probe	5’-TGACAACCACGGCCTTCCCTA-3’
IDO1	Forward	5’-TGAGCACGGACGGACTGA-3’
	Reverse	5’-GGCAGCACCTTTCGAACATC-3’
	Probe	5’-AGGTTACAGCGCCTGGCAC-3’
TDO2	Forward	5’-GACTGTCATACCGTGCACTC-3’
	Reverse	5’-GCGTGTCAATGTCCATAAGTG-3’
	Probe	5’-TCGATTCCAGGTCCCTTTCCAGC-3’
TiPARP	Forward	5’-CGGCTTGCCTGAGGATTGT -3’
	Reverse	5’-TCAGAAGGAGAAGGAGGCTGT -3’
	Probe	5’-ACCACTGAACCTGAGCCAGACTGT-3’
AHR(ex3-4)	Forward	5′-CAGGACCAGTGTAGAGCACA-3′
	Reverse	5′-CCATTCAGCGCCTGTAACAA-3′
	Probe	5′-ACTCTCCTTCTTGCAAATCCTGCCAGT-3′
AHR(ex1-2)	Forward	5′-GCCAACATCACCTATGCCA-3′
	Reverse	5′-GTCTCTGTGTCGCTTAGAAGG-3′
	Probe	5′-CAGAAAACAGTAAAGCCCATCCCCG-3′
AHR cDNA	Forward	5’-ATGAGCAGCGGCGCCAACATCA-3’
	Reverse	5’-TCAACTCTGCACCTTGCTTAGGAATGCCT-3’

Supplemental table 1. Primers used for qRT-PCR

Supplemental Table 2

subset	CD45	CD11b	CD11c	CD24	CD64	MHCII	SiglecF	CD103	Ly6g	Ly6c	Thy1.2
cDC1	+	-	+	+	-	+	-	+	-	-	-
cDC2	+	+	+	+	-	+	-	-	-	-	-
Alv. Macs	+	-	+	-	+	+	+	-	-	-	-
Int. Macs	+	+	+	-	+	+	-	-	-	-	-
T.R. Monos	+	+	+	-	-	-	-	-	-	-	-
Inf. Monos	+	+	-	-	-	-	-	-	-	+	-
Lymphocytes	+	-	-	-	-	-	-	-	-	-	+
B-Cells	+	-	-	+	-	+	-	-	-	-	-
Granulocytes	+	+	-	+	-	-	-	-	+	-	-

Supplemental table 2. Markers used for identification of lung leukocyte populations by flow cytometry.

Supplemental Figure 3

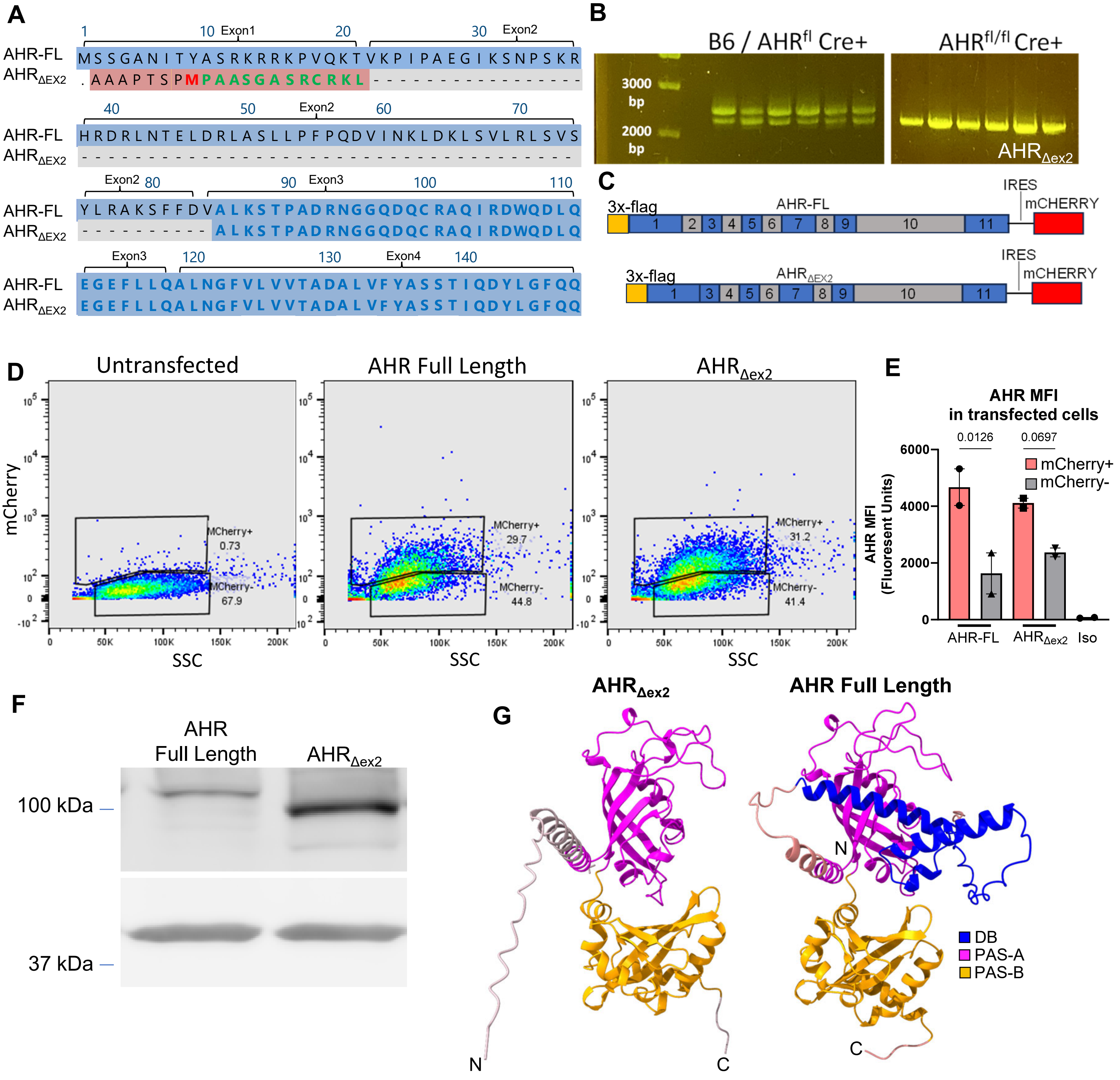

Supplemental Figure 3. Generation of CD11c-Cre / AHR$^{fl/fl}$ mouse (AHR$_{\Delta ex2}$) **and characterization of a truncated AHR product lacking exon 2.** (A) Schematic detailing the exon1-3 fusion that is produced in AHR$_{\Delta ex2}$ mice. Deletion of AHR exon 2 shifts the open reading frame revealing an alternative start codon (red text) which results in translation of a 12 amino acid leader sequence (green text) and exons 3 – 10 in frame (blue text). Dashed sequence represent the deleted amino acids of exon 2 in comparison to the full length native AHR sequence (AHR-FL). (B) PCR of AHR CDNA generated from iCD103 DCs from either B6 / AHR^{fl} or AHR$_{\Delta ex2}$ mice. (C) Schematic detailing the AHR-mCherry constructs that were created for in vitro expression. (D and E) NIH 3T3 cells were transfected with the indicated clone and analyzed for mCherry expression by flow cytometry in panel C or expression of AHR in panel C (AHR-FL = AHR full length, AHR$_{\Delta ex2}$= CD11c-Cre+ AHR$^{fl/fl}$, iso = isotype control). F) NIH 3T3 cells were transfected with the indicated clone and analyzed for expression of AHR via western blot with a C-terminal specific AHR antibody (top panel) or a control antibody against β-actin (bottom panel). G) Presumed structures of both full length AHR (right) and AHR$_{\Delta ex2}$ were predicted using AlphaFold showing the DNA binding domain (DB) and the two major protein interacting domains PAS-A and PAS-B.

Supplemental Figure 4

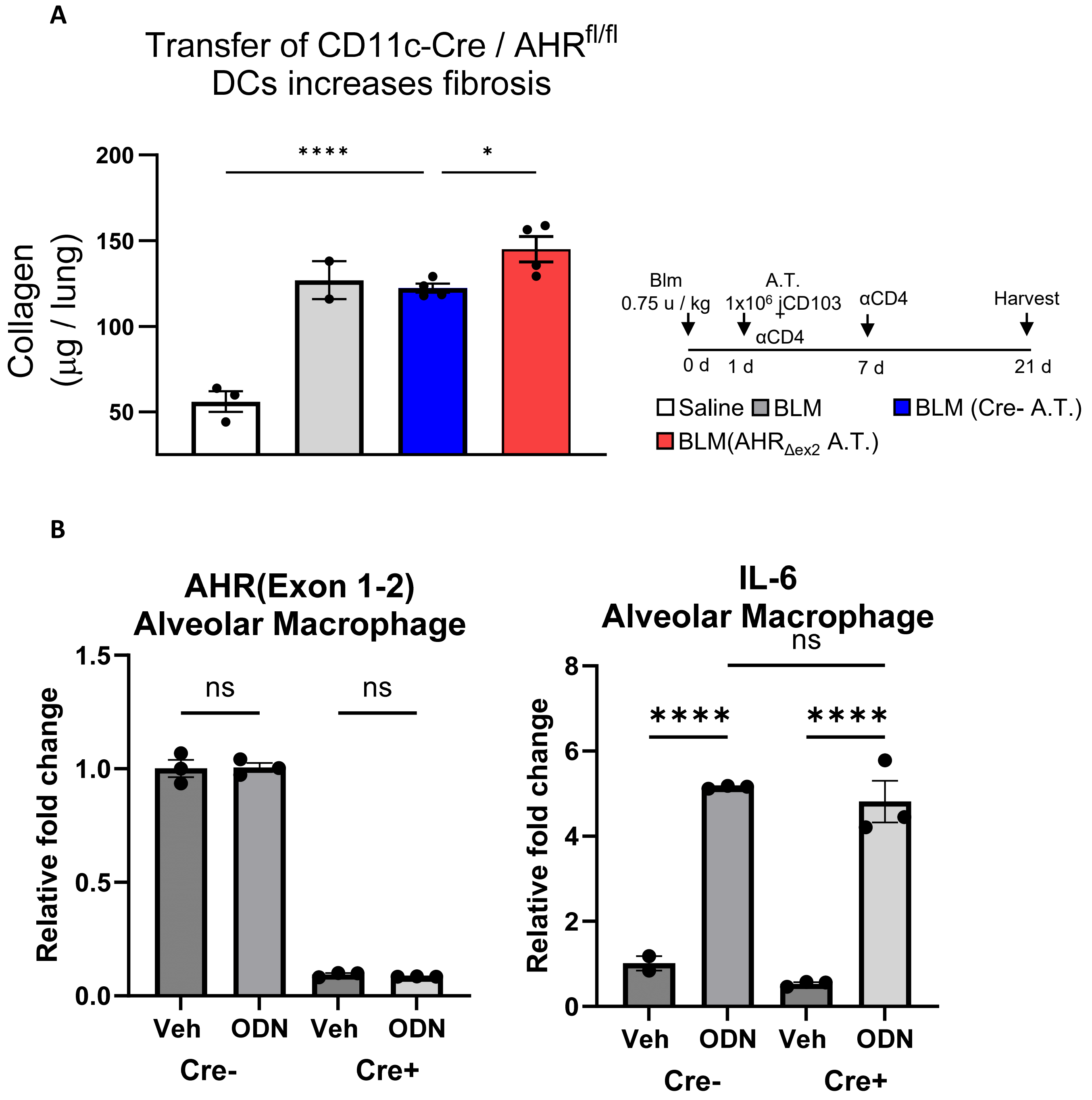

Supplemental Figure 4. Adoptive transfer of AHR$_{\Delta ex2}$ iCD103 DCs increases blm induced fibrosis in comparison to littermate control cells but alveolar macrophages do not exhibit AHR dependent IL-6 production. A) Mice (n = 2-4 per group) were administered 0.75 U/kg blm. At 1-d post BLM mice were given an adoptive transfer of 1x10^6 Cre+ AHR$_{\Delta ex2}$ iCD103 DCs (AHR$_{\Delta ex2}$ A.T.), Cre- littermate control iCD103 DCs (Cre- A.T.) or a sham transfer of PBS (BLM) via tail vein injection. Lungs were harvested 21-days post BLM treatment and collagen was quantified via hydroxyproline assay. Statistical significance was determined via ANOVA. **B)** Primary alveolar macrophages were isolated via bronchial alveolar lavage from either AHR$_{\Delta ex2}$ (Cre+, n = 5) or littermate control mice (Cre-, n = 5) and treated with the TLR9 agonist ODN2395 (ODN) or vehicle control for 18 hours. RNA was harvested and expression of the indicated transcript was analyzed via qRT-PCR.

Supplemental Figure 5

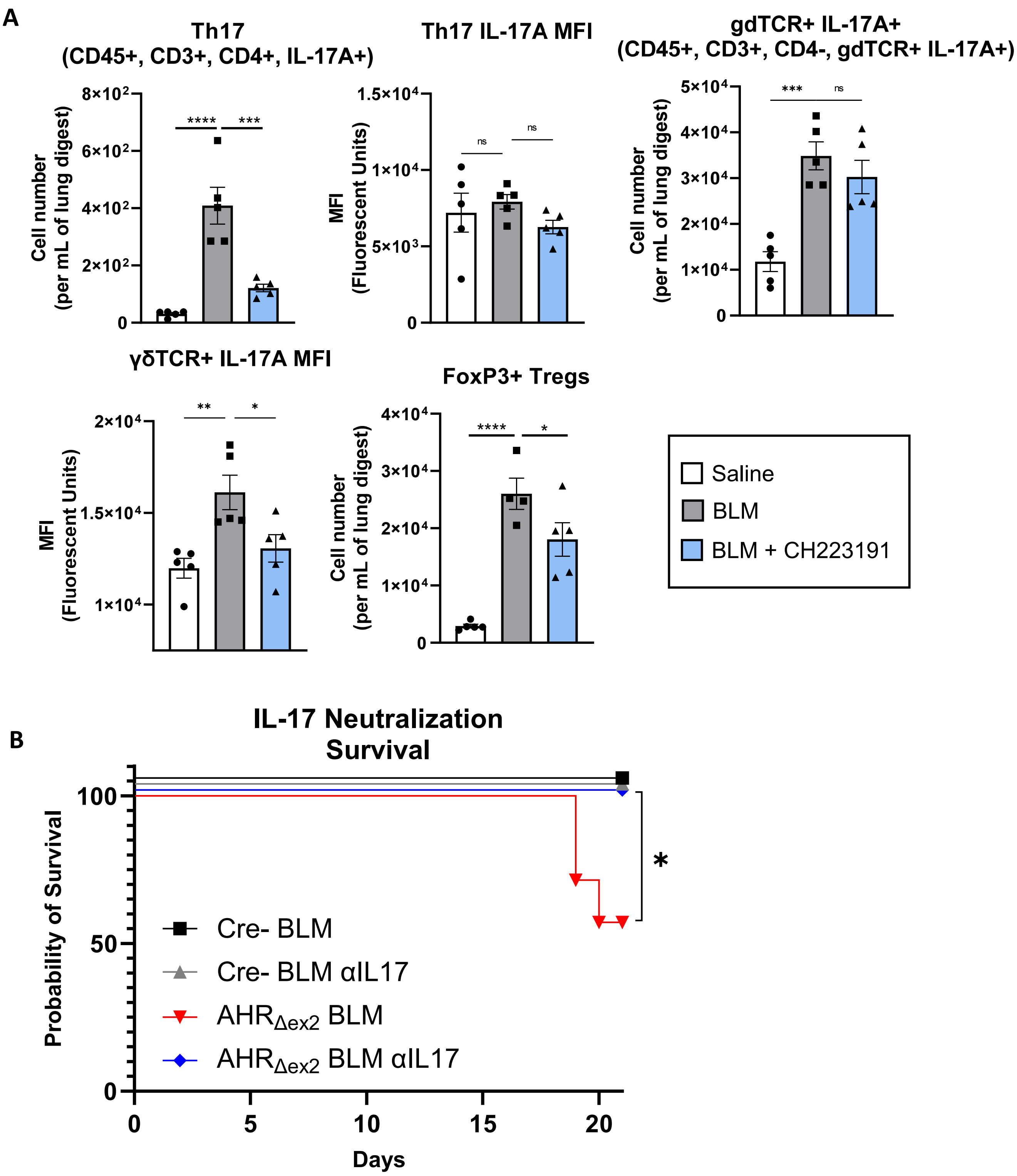

Supplemental Figure 5. AHR inhibition reduces IL-17 production in Th17 and γδ T-cells and neutralization of IL-17A protects from BLM induced mortality. A) Mice (n = 5 mice per group) were treated with BLM and lungs were harvested at 7-d post challenge. Flow cytometry was conducted on single cell suspensions to identify the following lymphocyte populations Th17 (CD45+, CD3+, CD4+, IL-17A+), IL-17 expressing γδ T-cells (CD45+, CD3+, CD4-, γδ TCR+, IL-17A+), and FoxP3+ Tregs (CD45+, CD3+, CD4+, FoxP3+). Statistical significance was determined via ANOVA. **B)** Mice (n = 5 – 7 per group) were treated with 0.75 units / kg BLM. At 3-d post BLM the indicated groups were treated with 100μg α-IL17 neutralizing antibody via i.p. injection. The remaining groups were treated with PBS control. Booster injections were given every 7 days and mice were harvested at 21-d post BLM.

Supplemental Figure 6

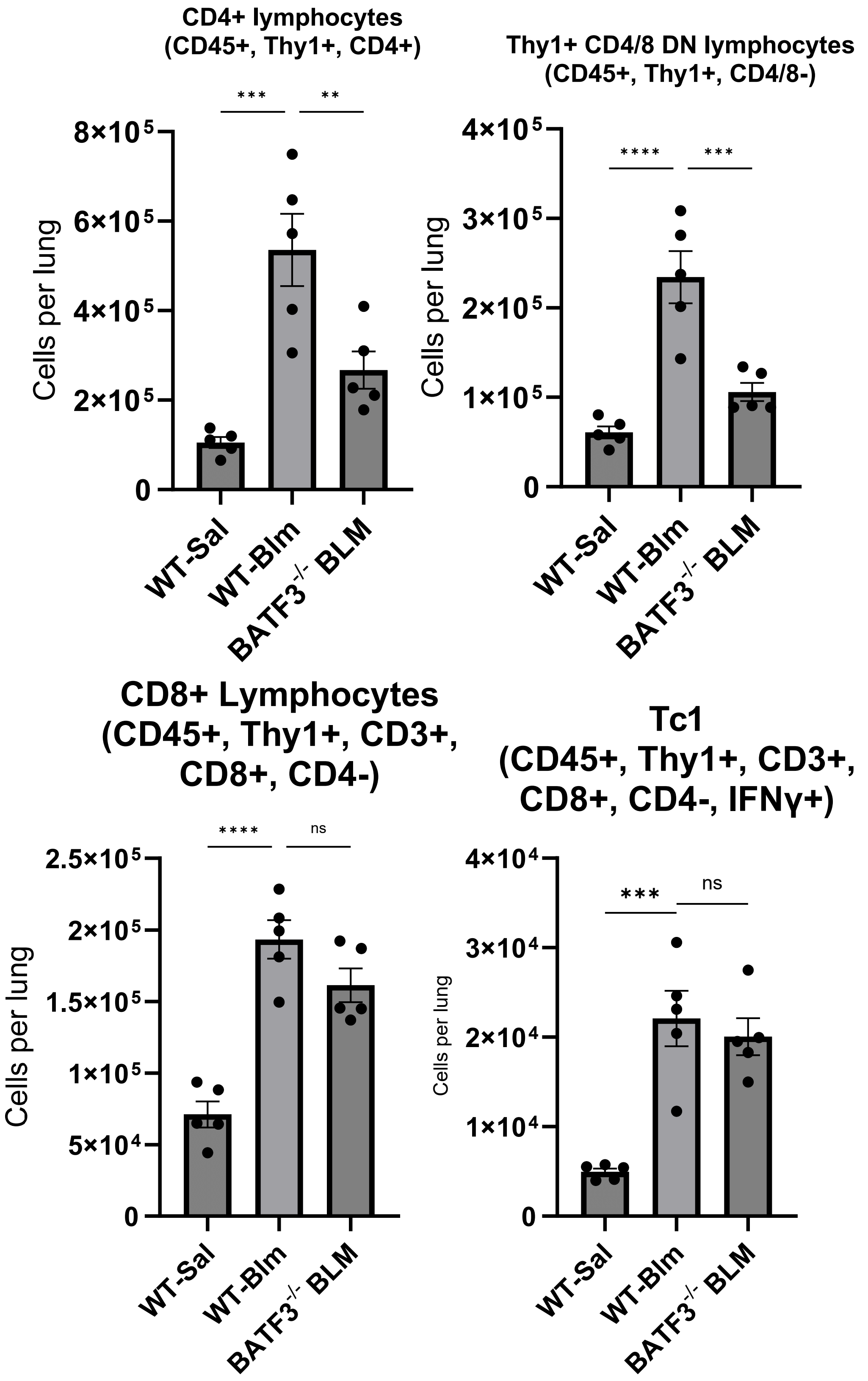

Supplemental Figure 6. BATF3-/- mice show no decrease in lung CD8 or Tc1 numbers following BLM challenge. Mice (n -5 mice per group) were treated with BLM and lungs were harvested at 14-days post challenge. Flow cytometry was conducted on single cell suspensions to identify the following lymphocyte populations Tc1 (CD45+, Thy1+, CD3+, CD8+, IFNγ+), IL-17 expressing γδ T-cells (CD45+, CD3+, CD4-, γδ TCR+, IL-17A+), and total CD8+ (CD45+, Thy1.2+, CD3+, CD8+). Statistical significance was determined via ANOVA.

Supplemental Figure 7

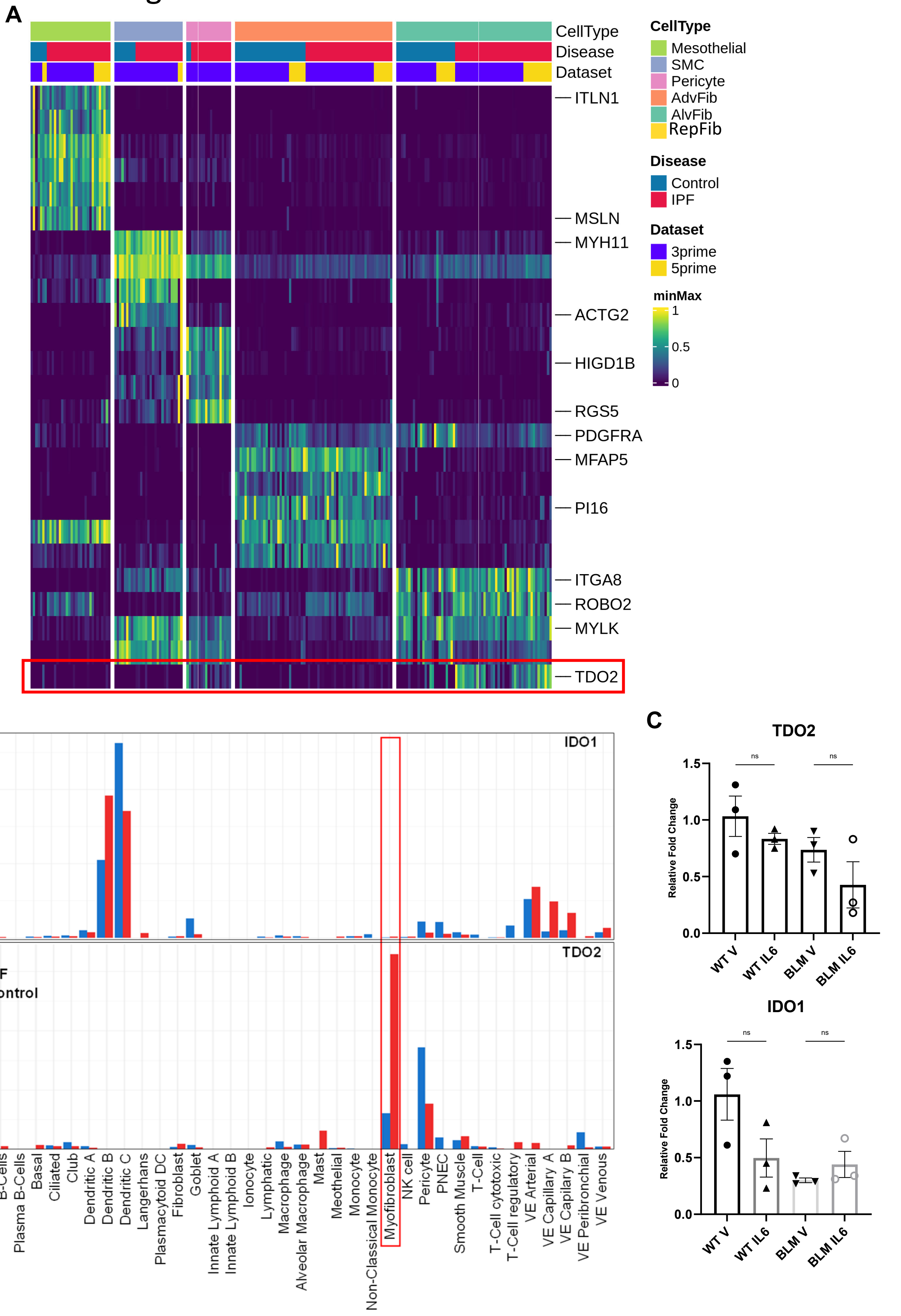

Supplemental Figure 7

Supplemental Figure 7. Myofibroblasts express TDO2 in the fibrotic lung and expression is consistent across multiple datasets. A) Heatmap of gene expression values across human lung stromal cells. Each column represents the average normalized expression of one cell type and one subject. Within each dataset, expression values for each gene are min-max scaled to the range of 0-1. **B)** Expression analysis of IDO1 or TDO2 displayed by cell type and disease state (Control samples = blue bars, IPF samples = red bars) taken from Adams et al. GEO ascension GSE136831. **C)** Lung fibroblasts were cultured from groups of mice treated with BLM for 21-d or untreated as control. Fibroblasts were subsequently treated with the 10 nM recombinant IL-6 for 18-h. Expression of the indicated transcript was analyzed via qRT-PCR. Statistical significance was determined via ANOVA.

Supplemental Figure 8

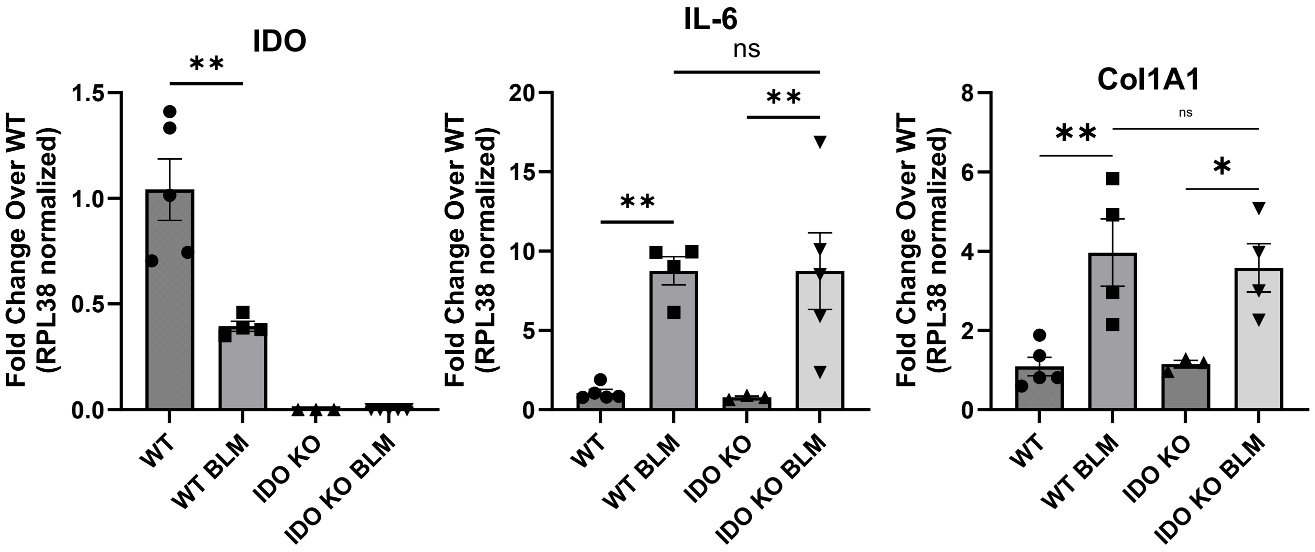

Supplemental Figure 8. Genetic deletion of IDO1 is not protective from blm induced fibrosis. Mice (n = 3-5 mice per group) were treated with BLM and lungs were harvested at 21-days post challenge. RNA was isolated from whole lung and analyzed via qRT-PCR for the indicated transcript. Statistical significance was determined via ANOVA. * = P < 0.05, ** = P < 0.01.

Supplemental Figure 9

A

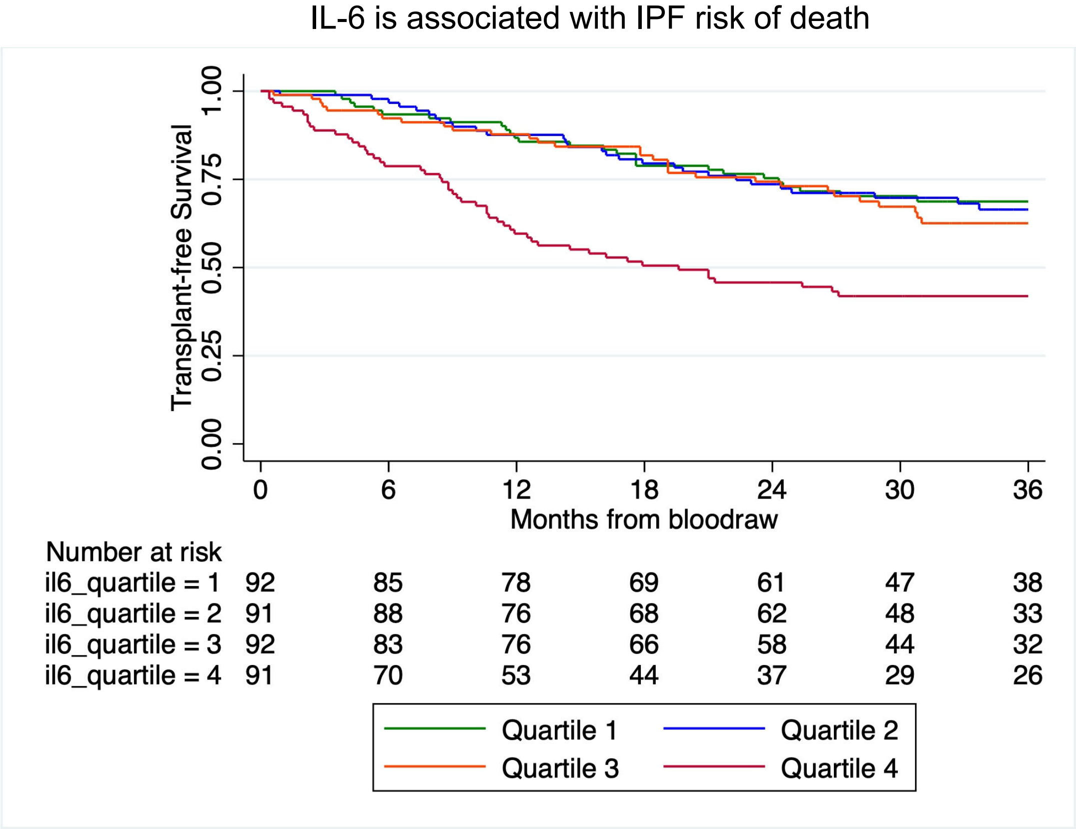

B

Risk of death or lung transplant within 36-months of blood draw			
**Biomarker**	**HR**	**95% CI**	**p-value**
IL6	1.41	1.24-1.61	<0.001
IL17a	0.98	0.87-1.12	0.83
IL17c	1.26	1.08-1.48	0.004
IL17d	0.55	0.36-0.83	0.005
IL17f	1.26	1.06-1.50	0.007

C

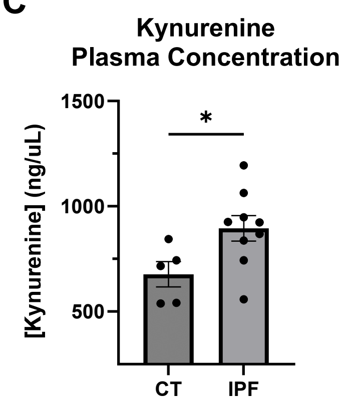

Supplemental Figure 9. Plasma concentrations of IL-6 and IL-17 isoforms are associated with risk of death or transplant in IPF patients. A) Survival curve showing correlation between IL-6 expression and transplant free survival in a cohort of IPF patients (n = 366). **B)** Table showing hazard ratios for IL-6 and Il-17 isoforms in IPF patients. **C) P**lasma from either IPF patients (IPF, n = 9) or healthy control (CT, n = 5) was analyzed for the concentration of kynurenine via ELISA. Statistical significance was determined via t-test * = p < 0.05.

Supplemental Methods.

Mouse line, cell lines, and reagents. WT C57Bl/6J mice were ordered from Jackson labs and used between 6-8 weeks of age. $AHR_{\Delta ex2}$ mice were generated by crossing the AHR^{fx} line (Ahrtm3.1Bra/J, strain 6203, Jackson labs) with a CD11c-Cre expressing line (B6.Cg-Tg(Itgax-cre)1-1Reiz/J, strain 8068, Jackson labs). Resulting pups were verified to contain both Cre and the AHR floxed alleles via PCR using Jackson labs recommended primers. CD11c+ cells were further verified to contain the AHR exon2 excised allele via PCR using Jackson labs recommended primers. $AHR_{\Delta ex2}$ mice were bred at the University of Michigan mouse breeding facility and used between 6-8 weeks of age. For some experiments AHR^{fx} mice which did not express CD11c-Cre were used as littermate controls. IDO1 knockout mice were purchased from Jackson labs (005867, B6.129-Ido1tm1Alm/J). Bleomycin was purchased as Blenoxane from the pharmacy at the University of Michigan hospital, 1U/mL stocks were made using sterile saline and mice were administered 0.75U / kg via the oropharyngeal route. The AHR agonist Kynurenine was used at 40mg/kg for *in vivo* experiments. The AHR and TDO2 inhibitors CH223191 and 680C91 (Tocris) were used at 5 mg/kg and 15mg/kg respectively for *in vivo* experiments, CH223191 was used between 10-30 µM for *in vitro* treatment of cell culture. Importantly, C80C91 is poorly soluble in aqueous buffer, thus, a solubilization buffer of 4% Tween-20, 0.5% Carboxy-Methyl-cellulose in PBS was used for *in-vivo* delivery. When properly solubilized, solutions will appear orange and transparent. Poorly solubilized solutions will appear milky and off white and exhibit lessened efficacy of the drug. We have also noted differences in solubility between vendors with drug obtained from Tocris showing the best solubility and other vendors, such as MedChemExpress, showing very poor solubility. The TiPARP (PARP7) inhibitor RBN2397 (Selleckchem) was used at a concentration of 10 µM. The TLR9 agonist ODN2395 (Invivogen) was used between 0.1 – 1.0 µM depending on cell type. The IL-17A neutralizing antibody (clone 1743, BioXCell) was given at 100 µg every 7-days via i.p. injection.

Generation of inducible iCD103+ DCs. Bone marrow cells were aseptically isolated from the femurs of indicated mouse lines and incubated for 16-days in RPMI media containing 10% heat inactivated FBS, 1% Pen/Strep cocktail, 50µM β-mercapto-ethanol, 3 ng/mL GMCSF (R&D systems) and 200 ng/mL Flt3-L (R&D systems) before any additional treatment. iCD103 cells were characterized to be CD11c+, MHCII+, and CD103+ by flow cytometry. For adoptive transfer experiments, Groups of WT-B6 mice were first administered BLM before receiving

1x10^6 iCD103+ cells from $AHR_{\Delta ex2}$ mice via tail vein injection. CD4 T-cell depletion was carried out via i.p. injection of 100 µg anti-CD4 depleting antibody (clone GK1.5 Bioxcell) at 0- and 7-days post BLM instillation [1].

Single Cell RNA-seq analysis. Read 2 poly(A) and template-switch oligo contaminants were trimmed using cutadapt (version 3.1). Reads were aligned to genome GRCh38 with STAR (version 2.7.8a) using Gencode annotation release 38.

Control and IPF cells previously classified as stromal were filtered from each dataset and combined in R (version 4.0.3) using the library Seurat (version 4.3.0.1). For normalization, raw expression counts were scaled to 10,000 transcripts per cell then natural log transformed with a pseudocount of 1. Cells were split by dataset and integrated using Seurat's reciprocal principal component analysis (RPCA) implementation, followed by dimension reduction, graph embedding, high granularity clustering and cell type re-annotation. These steps were performed iteratively until all multiplets were identified and removed.

Of the filtered data presented: Habermann et al. consists of 2,801 cells from 10 controls and 12 IPF lungs; Adams et al. consists of 6,850 cells from 26 control and 32 IPF lungs. Integrated gene expression values were only used for PCA-based graph embedding; cluster interrogation and subsequent visualizations were performed with normalized, empirical values. For the heatmap, normalized gene expression values of each cell type were averaged for each subject with 3 or more respective cells. To avoid batch effect between datasets when visualizing gene expression, normalized expression values were further min-max normalized across cells within each dataset.

Generation of single cell suspensions and flow cytometry. Mouse lungs were first perfused via injection of 3-5mL of saline through the right ventricle of the heart. Lungs were resected and minced with scissors before incubation in a collagenase buffer containing 1 mg/mL Collagenase-A (Roche), 2000U DNaseI (Sigma), in complete DMEM media supplemented with 10% heat inactivated FBS and 1% Pen/Strep cocktail for 45 minutes at 37°C in a shaking incubator. Cell suspensions were disrupted by drawing through a 10 mL syringe before filtration through a 100 µm pore sized cell strainer. Resulting single cell suspensions were assayed for cell viability via trypan blue exclusion.

Staining for flow cytometry was carried out via incubation with appropriately diluted fluorophore conjugated antibodies. Antibodies used for flow cytometry were as follows: Myeloid flow cytometry staining panel, BV650-CD11b (clone M1/70), BV510-CD45 (clone 30-F11), BV421-I-

Ab (MHCII clone AF6-120.1), APC-Cy7-SiglecF (clone E50-2440), APC-Ly6G (clone 1A8) purchased form BD Horizon. BV605-CD64 (clone X54-5/7.1), PerCP-Cy5.5-CD24 (clone M1/69), PE-Cy7-Ly6C (clone HK1.4), PE-CD193 (CCR3 clone J073E5) purchased from Biolegend. PE-eFluor610-CD11c (clone N418), PE-AHR (clone 4MEJJ) purchased from eBioscience. Lymphocyte flow cytometry staining panel. PE-Cy7-IFNγ (clone XMG1.2), FITC-CD3 (clone 17A2) purchased from BD bioscience. APC-Cy7-IL17A (clone TC11-18H10.1), BV570-CD8a (clone 53-6.7), AF700-CD90.2 (Thy1.2 clone 30-H12), BV510-CD4 (clone GK1.5) purchased from Biolegend. PE-eFluor 610-FoxP3 (clone FJK-16s) purchased from eBioscience. For intracellular detection of cytokines, single cell suspensions were first incubated in complete DMEM media with 10 % FBS and 10 ng / mL PMA, 10 µM ionomycin and Golgistop reagent (BD bioscience, concentration was used as per manufacturer recommendation) for 4 h at 37C. Cells were subsequently treated with a fixation and permeabilization kit (FoxP3 transcription factor staining buffer set following manufacturer recommendation, ebioscience) and subsequently incubated in appropriately diluted antibody cocktail. Note: Some markers were stained for but were not graphed. Flow analysis was carried out in FlowJo v. 10.5.

Isolation of CD11c+ DCs from single cell suspensions. CD11c+ cells were isolated from single cell suspensions using CD11c ultrapure microbeads (Milltenyi) following the manufacturer’s protocol. Alternatively, CD103+ DCs were isolated from collagenase digested lung cells using a two-step magnetic bead separation. Total CD11c+ cells were first isolated using CD11c-PE conjugated primary antibody in conjunction with a PE-Release magnetic bead (Stemcell technologies) after release of the anti-PE magnetic bead, a second CD103-FITC primary antibody was added and CD103+ cells were isolated using anti-FITC magnetic beads (Stemcell technologies) all beads were used in accordance with manufacturer recommended protocols.

Analysis of plasma concentrations of IL-6 and IL-17 isoforms. Circulating plasma concentrations for IL6 and IL17 (a, c, d and f isoforms) were determined used the Olink (Uppsala, Sweden) Explore platform, which uses proximity extension assays to generate semi-quantitative protein data, which are Log2 transformed for modeling. The association between plasma biomarker concentration and three-year transplant-free survival was assessed in a combined cohort of patients with IPF (n=366) from the University of Virginia, University of Chicago and University of California at Davis using univariable Cox proportional hazards regression [IRB approval and subject characteristics were previously reported (43)]. Survival

was plotted for each quartile of IL6 concentration using the Kaplan-Meier estimator. The proportional hazards assumption was checked and satisfied for each biomarker tested.

Quantification of collagen via hydroxyproline assay. Lungs from BLM or WT mice were resected at the indicated timepoints and homogenized in a buffer of PBS and complete protease inhibitor (Roche). Lung homogenates were diluted 1:1 with 12N hydrochloric acid and incubated overnight at 120°C in well-sealed glass tubes. The following day, aliquots of acid-hydrolyzed lung homogenate were incubated with a chloramine-T solution and then reacted with 4-(Dimethylamino)benzaldehyde. Hydroxyproline was detected via absorbance at 560 nM on a synergy H1 spectrophotometer (Biotek).

Isolation of RNA for qRT-PCR. RNA quantity and purity was assessed on a nanodrop spectrophotometer (Thermo). Transcript expression was analyzed via reverse transcriptase quantitative real-time PCR using Lunaprobe reagent (New England Biolabs) on a Quantstudio3 thermocycler (ABI). Expression analysis was conducted using a ΔΔct calculation. Transcripts were normalized to expression level of a housekeeping gene, RPL38, using a pre-validated primer probe set (Integrated DNA technologies, Hs.PT.58.40595235.gs). Other primer and probe sequences are detailed in Supplemental Table 1.

[1] Woroniecka KI, Rhodin KE, Dechant C, Cui X, Chongsathidkiet P, Wilkinson D, Waibl-Polania J, Sanchez-Perez L, Fecci PE: 4-1BB Agonism Averts TIL Exhaustion and Licenses PD-1 Blockade in Glioblastoma and Other Intracranial Cancers. Clin Cancer Res 2020, 26:1349-58.
