# Supplementary material for: CD103+ dendritic cell–fibroblast crosstalk via TLR9, TDO2, and AHR signaling drives lung fibrogenesis
Source: JCI Insight. 2025 Feb 18;10(6):e177072. doi: 10.1172/jci.insight.177072 (PMC11949071; doi:10.1172/jci.insight.177072)
Supplement: Unedited blot and gel images [file jciinsight-10-177072-s193.pdf]

AHR 4MEJJ

Supplemental Figure 3, lanes 2 and 3 shown in paper. Other lanes show mutations not relevant to this manuscript.

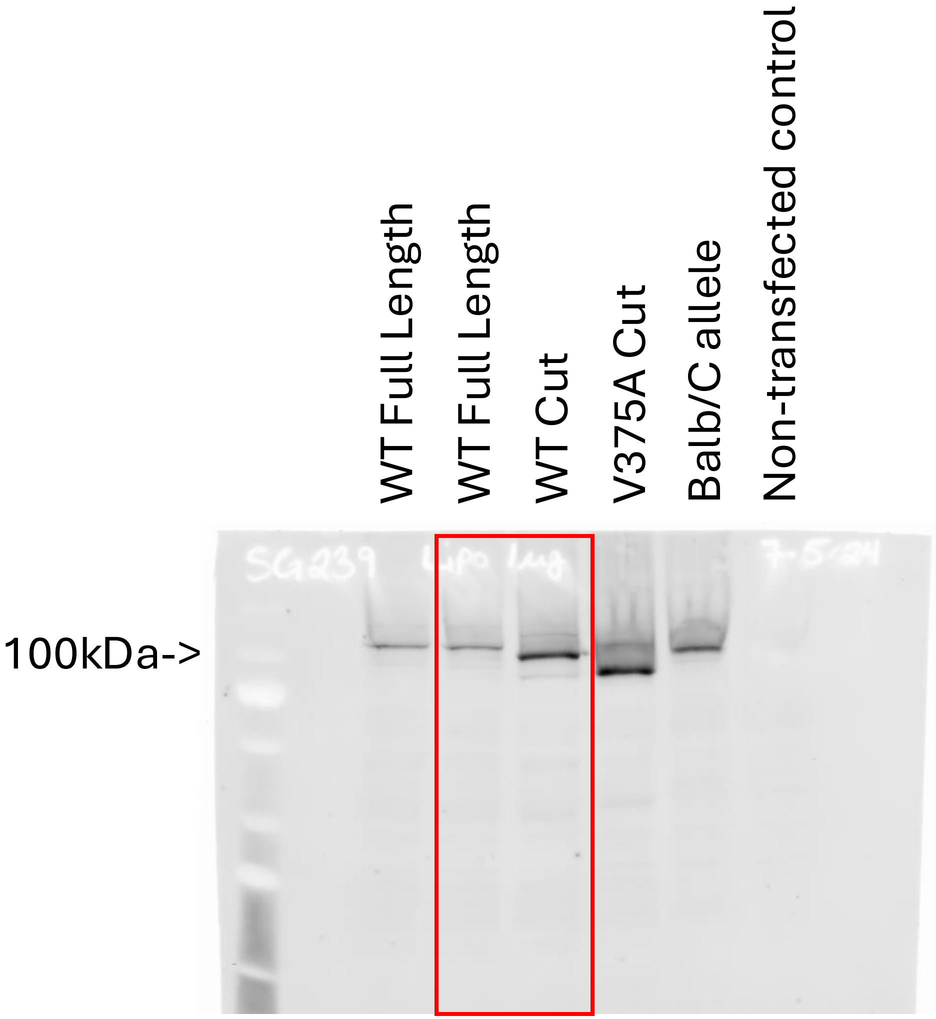

B-actin

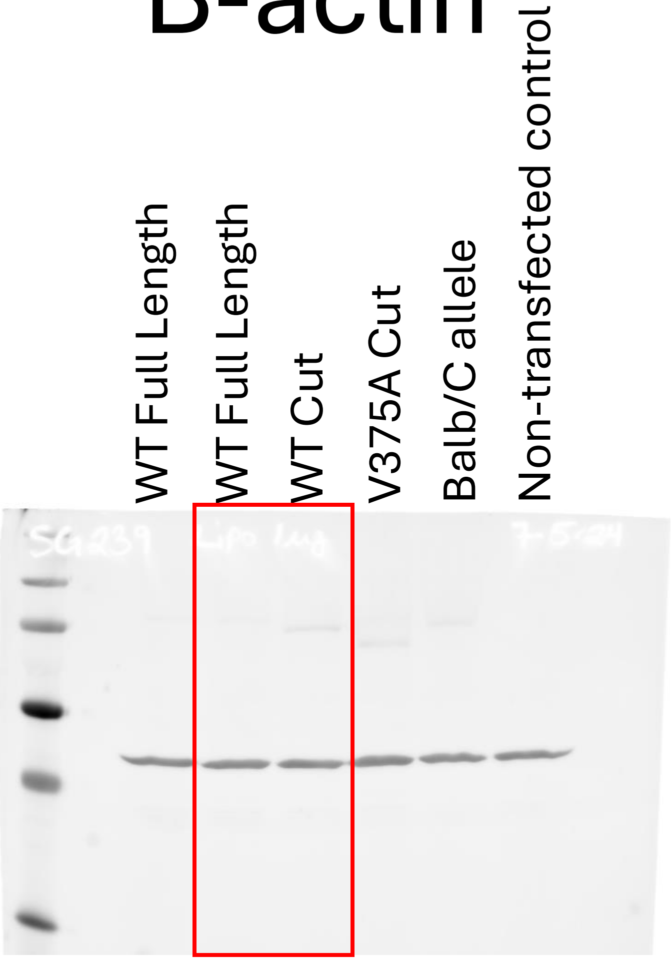

WT Full Length
WT Full Length
WT Cut
V375A Cut
Balb/C allele
Non-transfected control
